# Psychological Balance Scale: Validation Studies of an Integrative Measure of Well-Being

**DOI:** 10.3389/fpsyg.2021.727737

**Published:** 2021-09-16

**Authors:** Anastasia Besika, Andrea B. Horn, Mike Martin

**Affiliations:** ^1^Department of Psychology, University of Zurich, Zurich, Switzerland; ^2^University Research Priority Program “Dynamics of Healthy Aging”, University of Zurich, Zurich, Switzerland; ^3^Center of Competence in Gerontology, University of Zurich, Zurich, Switzerland

**Keywords:** psychological balance, flexibility, consistency, self/others ratio, well-being, meaning in life, satisfaction with life

## Abstract

Studies infer Psychological Balance from the absence of psychopathology. In this article, we investigated this construct as an antecedent of well-being. We present empirical evidence toward the validation of a new theoretical model regarding Psychological Balance, a dynamic state with relatively constant characteristics, comprising Consistency and Flexibility and influenced by a Self/Others Ratio. A battery of 31 items, as indicators of Consistency, Flexibility, and Self/Others Ratio, aided this empirical investigation. In an online study (*N* = 933), we collected cross-sectional data from the United Kingdom. Results of cross-validation analyses provided evidence toward the validity of the proposed model and the psychometric properties of its instrument. There were statistically significant associations between Consistency (i.e., degree of integration of a universal value structure as self-related characteristics that motivate personal goals and behavior), Flexibility (i.e., degree of ability to re-define meaningful and important goals in response to situational challenge), and five well-being variables (e.g., Meaning in Life). Self/Others Ratio (i.e., ratio of motivation to serve self-interest and the interest of others), operationalized as a binary variable (e.g., close and away from 1), moderated some of these associations. Altogether, this work may contribute toward a nuanced understanding of well-being and form the basis of interventions that aim to decrease emotional discomfort and increase meaning, happiness, and life satisfaction.

## Introduction

In the literature, absence of psychopathology infers *Psychological Balance*, which is associated with psychological continuity across time (Fraley and Roberts, 2005) and an ability to cope with daily challenges (Nielsen and Knardahl, 2014). In spite of indirect references to a strong association between Psychological Balance and well-being, investigations of how an individual remains psychologically stable in a constantly changing environment are missing. The present article addresses this gap and provides an instrument for assessing *Psychological Balance* that refers to a dynamic psychological state with relatively constant characteristics, comprising *Consistency* and *Flexibility*. The integrative model of Psychological Balance provides a unifying theoretical framework that connects emotion, cognition, and behavior. In addition to the theoretical model, we present the development and first validation studies of a multi-dimensional instrument that serves as an indicator of Psychological Balance. Results of a cross-validation study support our theoretical assumptions and the model that is concerned with the antecedents of well-being and the psychological mechanism that allows individuals to maintain psychological stability while adapting to situational and developmental change. In addition, we provide an instrument for assessing Self/Others Ratio, a novel factor that influences the relationship between Psychological Balance and well-being. We present evidence that the ratio of motivation to serve *self-*interest and the interest of *others* (*Self/Others Ratio*) moderates the relationship between Psychological Balance and well-being variables. These instruments carry the potential of identifying the areas that undermine Psychological Balance (e.g., inconsistent behavior due to low levels of value awareness) and target it with effective interventions.

### Concepts of Balance

Aristotle (385-323 BC) taught that *balance* is the key to a happy and meaningful life, found in the “golden mean” between excess and deficiency. For example, being benevolent balances the two opposites of envying others and self-sacrifice (Ross, 1956). This philosophical perspective associates balance with personal engagement in discovering a set of pre-existing principles and adopting them as personal virtues. A psychological perspective associates the idea of balance with integrating multiple life roles as parts of self-identity and operating within multiple life domains. Studies indicate that an increased capacity for integrating multiple roles into the self-concept is associated with high self-esteem and low depression (Marks and MacDermid, 2006). However, when investigating well-being researchers typically focus on the association of socio-demographics, personal characteristics and values with positive affect and life satisfaction (e.g., Lucas et al., 2004; Schwartz and Sortheix, 2018), which are outcome variables. Although more recently, the research on meaning extended the knowledge on what contributes to well-being (King and Hicks, 2021), questions concerning the psychological structure that strives to balance in a spatio-temporal environment remain unanswered. Assuming a negative feedback loop mechanism that regulates behavior (Carver and Scheier, 1981) and maintains a person’s happiness at a relatively stable level overtime, regardless of life events (Heady and Wearing, 1992), what are the psychological processes that contribute to their emotional equilibrium? The model of Psychological Balance is concerned with the gap in addressing the psychological antecedents of well-being. The proposed model combines an Aristotelian perspective with the idea that people need to integrate multiple qualities in order to respond to life demands (Nordenfelt, 1993; Marks and MacDermid, 2006).

### An Integrative Approach to Balance

In this article, we investigated the theoretical assumptions that Psychological Balance is influenced by *Consistency* that provides stability, and *Flexibility* that facilitates change. *Consistency* refers to the integration of a universal value structure (Schwartz, 1992) as a pattern of self-related characteristics that influences personal goals and manifests in behavior patterns. Research findings suggest that congruence of personal goals and values is positively associated with subjective well-being, measured as global life satisfaction using the Satisfaction with Life Scale (SWLS) (Palys and Little, 1983; Diener et al., 1985; Sheldon and Kasser, 1995). Although there are measures that assess the importance of social values to the individual (e.g., Schwartz’s Value Survey; Schwartz, 1992), these focus on single values and do not assess the degree values influence people’s motivation, goals, and behavior as a system. *Flexibility* refers to the cognitive ability to re-adjust value priorities in response to change. Research evidence indicates that re-adjusting personal preferences and goal orientation, as a way of maintaining a positive perspective during highly adverse circumstances, is positively associated with life satisfaction and is negatively associated with depression (Brandtstädter and Renner, 1990; Odacı and Cikrikci, 2019). The various instruments measure aspects of cognitive flexibility they are concerned with that goal adjustment. However, their focus is not on value-related goals. Many items among these instruments are semantically identical and may be reduced to a more concise scale, for example, the item “I think about other goals to pursue” from the Modification of Goal Adjustment Scale (Wrosch et al., 2003) and “I look for a new goal” from the Loss-based selection section of the SOC scale (Baltes et al., 1999). Thus, to test our theoretical assumptions required the development of a new instrument to help demonstrate empirically a positive relationship between Consistency, as well as Flexibility, and theoretically relevant well-being variables, as a validation for the proposed model. Another theoretical assumption is that as a dynamic state, Psychological Balance can bifurcate under certain conditions (Arnold et al., 2013). Assuming that the two mental contexts of *self* and *others* underlie the self-concept (Higgins, 1987) and a dual motivation to serve personal and others’ interest underlies social values (Schwartz, 1992), we postulate that beyond a level of self-discrepancy (Higgins, 1987), where the *self* and *others* are integrated at a significantly different degree from each other, the psychological equilibrium becomes unstable. In other words, when values motivate an individual to serve their personal interest significantly more than to serve the interest of others, they are likely to be less flexible in adapting to situations that demand placing their focus on other people (Gaertner et al., 2008). As a result, the individual is likely to experience distress and difficulty in adaptation. When values motivate an individual to serve the interest of others significantly more than serving their personal interest, this is likely to undermine their autonomy and self-development (Chirkov et al., 2003). Given the novelty of the construct, testing this assumption required the development of two items, one that represents the nominator and one the denominator of the Self/Others Ratio. Furthermore, identifying a *self* to *others* ratio range that signifies differences in well-being measure scores would validate the assumption of bifurcation. In the following, we introduce the theoretical background and empirical findings that informed our proposed model.

#### Value Pattern and Consistency

**FIGURE 1:**
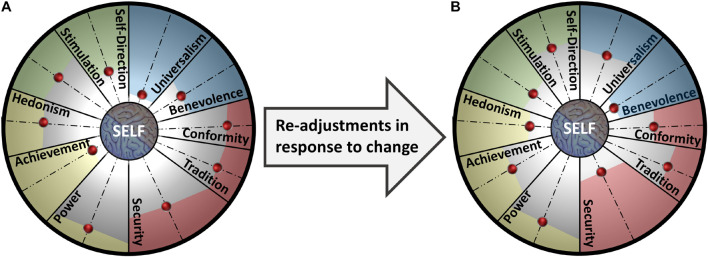
**(A)** Value pattern within the value structure. **(B)** Value pattern fluctuation in response to change.

Over the last 30 years, studies have been showing that a set of universal values serve, to a different degree, as ideals that guide people’s goals and behavior (Schwartz, 1992; Schwartz and Cieciuch, 2021). The most parsimonious model of the 10 value domains, including Self-Direction, Stimulation, Hedonism, Achievement, Power, Security, Conformity, Tradition, Benevolence, and Universalism, represents values in a circular continuum (Schwartz, 1992). This circular structure is interpreted as a representation of the relationship between values, where adjacent values are considered complimentary and values opposite each other to be conflicting. A longitudinal study with children 7–11 years of age indicates that this value structure begins to formulate at an early stage of development as a cognitive pattern that influences behavior (Cieciuch et al., 2016). Experimental studies suggest that values behave as a pattern. In an experiment, endorsing the value of health activated a range of other values. Participants reported that to value health required to embrace responsibility, strength, family, and helpfulness (Allicock et al., 2008). In another investigation, activating a single value that was central to a person’s self-concept caused other values that were less important to the participants to influence their behavior. The closer a value was to the self-concept, the stronger it influenced the person’s identity and behavior. The further away a value was held from the self-concept, the weaker its influence was on their identity and behavior (Verplanken and Holland, 2002; Verplanken and Sui, 2019). This evidence suggests that values operate as a pattern that is structured in relation to the self, which moderates the relationship between values and behavior. Overall, these finding indicate that people integrate a universal value structure as a set of self-related criteria that aid self-evaluations in relation to the external world. To illustrate the relationship between the individual and the universal value structure, we conceptualized the self at the center of the circular continuum of the 10 value domains. Serving as the reference point during value integration, the self constructs a value pattern that denotes the importance each value holds for the individual at the time of integration. Figure 1A depicts the circular continuum of the 10 value domains (Schwartz, 1992) and the value pattern of an individual. The importance of each value to the individual is represented by a red (round) “switch.” The closer the “switch” is to the center of the structure, the more important the value is to the person. For example, Figure 1A may represent *Robert’s* value pattern that influences his goals and manifests in his behavior patterns. *Robert’s* value pattern may represent a person who is a successful businessman (Achievement) travels the world to learn new things (Self-Direction, Stimulation) and meets clients to gain an insight of their needs in order to develop products that benefit many people in the world (Universalism). *Robert* hardly sees his family, but he tries to earn good money and provide them with everything they need (Security, Benevolence). This example demonstrates that the value structure facilitates stability as it contributes to a person’s sense of identity and allows them to connect to their social context.

##### Values and well-being

Previous cross-sectional investigations of the relationship between the 10 value domains and well-being or behavior focus on the values people mostly prioritize. For example, the Schwartz Value Survey was used to investigate which values are mostly associated with well-being (e.g., Karabati and Cemalcilar, 2010). This line of research dichotomizes the circular continuum into “healthy” (or anxiety free) vs. “unhealthy” (or anxiety avoidance) values (Kasser and Ryan, 1996; Schwartz and Sortheix, 2018). Values that were associated with growth and self-actualization (e.g., Stimulation, Self-Direction, Universalism, Benevolence, and Achievement) are considered to enhance well-being and values that were associated with self-interest (e.g., Conformity, Security, and Power) are considered to undermine well-being (e.g., Bobowik et al., 2011). However, this conceptual division of values derives from a methodological approach where researchers typically prompt participants to rank or rate their value preferences, placing an emphasis on value hierarchy. Focusing on value priorities overlooks that values behave as an interrelated pattern and less prioritized values possibly play a role in a person’s behavior and well-being. Schwartz noted “people tend to rate all values relatively high or low, regardless of content” (Schwartz, 2016, p. 253). Could this indicate different levels of value integration of the value structure? We speculate that focusing on value priorities prevents the emergence of a broader picture about their systemic relationship to well-being.

In treating the 10 value domains (Schwartz, 1992) as a dynamic structure, we introduce a novel approach to investigating their relationship to well-being by focusing on the stability of the individual’s value pattern and its fluctuations across time. In this cross-sectional study, we aimed to investigate values as a stable pattern at one point in time, and investigating its fluctuations would require longitudinal studies. Our first task entailed developing a measure for Consistency using 10-fold items that assess the degree the 10 value domains constitute ideals that motivate goal pursuit and daily action. This study aims to examine the relationship between the value pattern, as it influences goals and action, and well-being. We expect this level of aggregation to describe a factor of high importance to an individual’s well-being.

#### Value Pattern and Flexibility

The model of Psychological Balance assumes that values change in importance and their pattern fluctuates in relation to the self, as a person’s internal and external worlds change across the life span. When the area in which people find meaning is threatened, people seek meaning in other meaningful domains (Heine et al., 2006). Thus, re-adjusting value priorities could aid adaptation to change. Studies show that in early stages of development, children predominately integrate Security, as it helps them adapt safely into the outside world and feel protected. As they grow older, children integrate more the value of Stimulation, as they become more interested in the outside world and are motivated to explore it (Cieciuch et al., 2016). Changing value priories allows a person to re-evaluate themselves and the external world and adapt their goal priorities and behavior to the new situation, either temporarily or more permanently. Evidence suggests that in response to change the individual’s value pattern fluctuates systematically. As the importance of one value decreases, the importance of other values increases (Bardi et al., 2009). For example, Figure 1B represents the re-adjustments *Robert* made in his value priorities in response to an unexpected life challenge. When his son had heart surgery, he canceled his business trip to New York and missed an important business meeting to be with his family. *Robert* realized that spending time with his wife and son was more important than creating useful products for the world. He decided to spend less time traveling and spend more time having fun with his family. Changing his value priority allowed *Robert* to re-define his goals and respond to his family needs but also allow him to respond to his need to be close to the people he loves most. *Robert* could also have fluctuated back to his previous value pattern after his son’s heart surgery should the need to be close to them did not arise from the situation. This example demonstrates that the value structure facilitates change by allowing a person to change their value pattern and adapt to the new conditions.

In converging with the view that cognitive flexibility precedes behavioral re-adjustments (Paulhus and Martin, 1988), we assume that re-adjustments in the value pattern always precede re-adjustments in goal and behavior change. A line of research indicates that when a situation prevents an individual from pursuing their goals, a person may re-adjust their goals and behavior to change the situation in a way that matches the way they think. However, when resources are limited, a person can re-adjust the way they think to match the situation (Brandtstädter and Greve, 1994). In assessing Flexibility, attempting to assess value re-adjustment directly would involve many limitations, due to the salient nature of values (Verplanken and Holland, 2002). Instead, when constructing a measure for Flexibility, we aimed to construct items that assess ability to re-adjust meaningful and important goals in response to situational change, assuming that people refer to their values when they talk about meaningful and important goals (Reker and Wong, 1988).

Drawing on the above theories and empirical evidence, the model of Psychological Balance posits that the value structure constitutes a dynamic structural component of the self-concept. Due to an underlying mechanism, values maintain structural consistency and provide stability as they align the self to its social context and connect to the other components of the self-concept (e.g., goals and behavior). Characterized by flexibility, the value structure facilitates adaptation as it allows an individual to re-adjust their value pattern in response to developmental and situational change.

#### Self and Others

In addition to social values endorsed by the broader environment (Rokeach, 1968), theories suggest that *others’* (e.g., a significant other or a broader society) expectations also influence the value integration process during a person’s development (Kelly, 1970; Turner, 2010; Lewin, 2013). Hence, the value structure has two underlying motivational orientations, namely, to serve *self* and *others*’ interest (Sagiv and Schwartz, 2000). Assuming that *self* and *others* construct an individual’s implicit eco-system, what is the influence of *self* to *others* ratio on the relationship between an individual’s value pattern and their well-being? Research evidence indicates that the relationship between values and well-being is moderated by factors such as the level of congruence between a person’s values and the values promoted by their environment. A study reports that certain values, previously categorized as “unhealthy” (e.g., Power), were associated with high levels of life satisfaction when the same values were endorsed in the participants’ environment (e.g., Musiol and Boehnke, 2013). Another study shows that the level of congruence between a person’s values and their activities influences whether these activities are satisfying. Activities associated with values categorized as “unhealthy” (e.g., Conformity) were satisfying to the degree they were in line with people’s personal values (Oishi et al., 1999). Studies also suggest that personal goals and actions are influenced by and depend on others to a degree (Palys and Little, 1983). Since *self* and *others* co-exist in the person’s cognitive environment, the proposed model assumes that *self* and *others* underlie the value pattern, and consequently, a Self/Others Ratio moderates the relationship between the two sub-constructs of Psychological Balance and well-being.

Drawing on theories and findings, we postulate that *self* and *others* generate dynamics that facilitate adaptation. According to the Self-Discrepancy Theory (Higgins, 1987), the *self* and *others* underlie a person’s self-concept. *Self* and *others* refer to mental contexts that represent personal reality (Maio, 2010) and are held in tension within a person’s self-concept (Freud, 1962). Experimental findings indicate that when a person focuses on self-interest, they tend to shift their focus away from social interest (Gaertner et al., 2008) and further evidence shows that perceived discrepancies between *self* and *others* result in cognitive re-adjustments that aim to restore internal consistency (Higgins, 1987). Shifting interest orientation between *self* and *others* is essential to psychological functioning as it serves to maintain a sense of coherence in the relationship of a person with the external world (Heine et al., 2006; Park, 2010). Assuming that the two mental contexts of *self* and *others* constitute a person’s mental environment and given that values serve as criteria for evaluating oneself in relation to the external world, we posit that a dual motivation to serve *self* and *others* can both underlie a single value and therefore all of the value domains (cf. Schwartz and Sortheix, 2018). For example, the value of *altruism*, that denotes serving *others’* interest, is also a source of pleasure and benefits *self*-interest (Batson and Shaw, 2009).

##### Self/Others Ratio

Being motivated to serving *self*-interest at an overall similar level to serving the interest of *others* may indicate absence of self-discrepancies. Based on previous findings that show that reduced self-discrepancies reduce negative affect (Higgins, 1987, 1997), we postulate that people with a high capacity to integrate a motivation to serve *self* and *others* experience high levels of well-being. In contrast, a significant difference in the dominance of *self* over *others* or vice versa may indicate increased self-discrepancies and have a negative impact on well-being. For example, a person may hold the value of Tradition as an ideal that motivates them to fulfill their perceived expectation of their parents to marry and have children. If Tradition does not carry a motivation that serves *self-*interest (e.g., to fulfill their need to belong), *others* dominate *self* and the person is likely to experience psychological discomfort and family conflict. Therefore, people with a close to 1 Self/Others Ratio would experience higher levels of well-being compared to those with ratio ranges away from 1. We also postulate that the relationship between Consistency, as well as Flexibility, to well-being would be stronger in people with a Self/Others Ratio close to 1 in comparison to those with a ratio away from 1. These assumptions are in line with further research findings showing that healthy functioning is associated with an optimum ratio of positive to negative thoughts and emotions, beyond which a person manifests symptoms of psychopathology (Garamoni et al., 1991). Assuming that self-discrepancies generate negative emotion (Higgins, 1987), *self* and *others* need to maintain a healthy ratio range beyond which the system bifurcates and the psychological equilibrium becomes unstable. The conceptualization of Self/Others Ratio suggests that reconciling individual interest and the interest of other people contributes to adaptation across the life span, an idea in line with world philosophies and psychological theories (Freud, 1962; Reker and Wong, 1988; Lu et al., 2001).

### The Present Work

**FIGURE 2:**
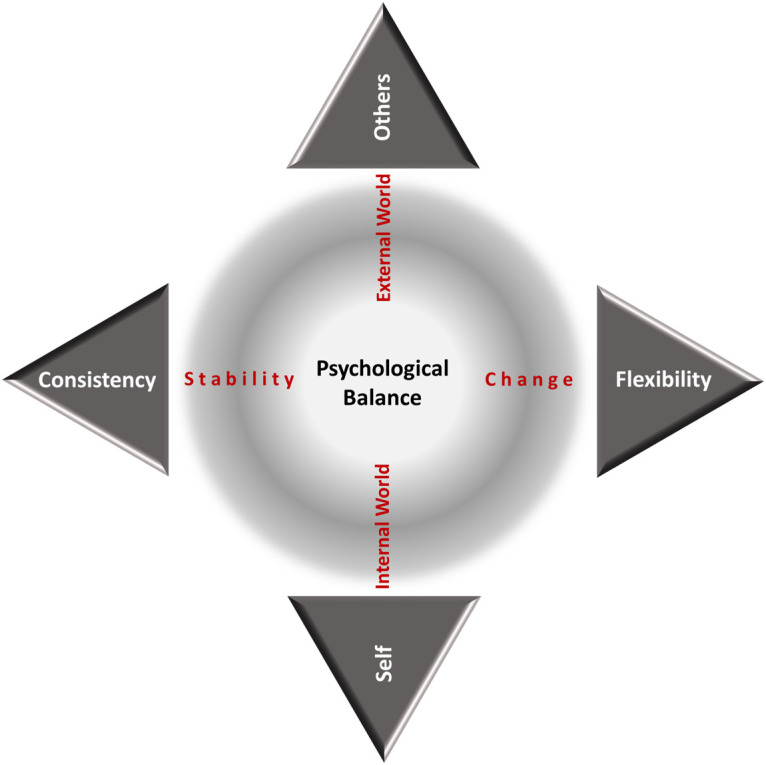
Psychological Balance moves on a horizontal axis between Consistency and Flexibility and on a vertical axis between Self and Others.

Overall, the proposed model suggests that people integrate a universal value structure as a unique value pattern that is underlain by a mechanism that facilitates: (a) stability through Consistency, by connecting what is most important and meaningful to people to their personal goals and actions; (b) change through Flexibility, by re-adjusting value priorities in response to situational and developmental change; and (c) positive affect though a Self/Others Ratio, by maintaining a range close to 1. Figure 2 illustrates Psychological Balance as the state where an individual’s level of Consistency and Flexibility reconciles their perceived internal and external worlds (*self* and *others*). The present study aims to provide measurement tools to test the validity of the proposed theoretical framework that addresses the following research questions: Does empirical evidence support the idea that Consistency and Flexibility are contributing factors to Psychological Balance? What is the range of Self/Others Ratio beyond which well-being decreases? To answer these questions, we aimed to fulfill three objectives: (i) develop valid and reliable measures for assessing Consistency, Flexibility and Self/Others Ratio; (ii) validate the proposed factorial structure of Psychological Balance; and (iii) identify a critical Self/Others Ratio range that influences the relationship between Psychological Balance and established measures of well-being.

## Materials and Methods

To test our theoretical assumptions, we first constructed a new measurement and then we administered the items that resulted from the content validation process in an online cross-sectional study.

### Scale Construction

In a two-stage scale development process, we first generated multiple items for each of the three constructs of interest, Consistency, Flexibility, and Self/Others Ratio and then followed a qualitative validation process (Kyriazos and Stalikas, 2018).

#### Item Generation

Assuming that values are not always salient in people’s awareness (Verplanken and Holland, 2002) and that meaningful goals are value-related (Reker and Wong, 1988), Flexibility was operationalized as the degree a person can re-adjust their most meaningful and important goals in response to change. Existing scales of Flexibility overlap with our conceptualization of the construct to a degree. Thus, in generating items for this subscale of Psychological Balance, we reviewed the literature and identified the following five inventories that assess different aspects of flexibility, aiming to extract the most relevant items: (1) The Loss-based Selection part of the SOC questionnaire (Baltes et al., 1999) includes 10 items that measure re-construction processes of goal hierarchy in response to decline or lack of resources. (2) The Flexible Goal Adjustment Scale (Brandtstädter and Renner, 1990) consists of 20 items measuring ability to change personal preferences as a way of maintaining a positive perspective. (3) The Cognitive Flexibility Scale (Martin and Rubin, 1995) consists of 12 items measuring willingness, and self-efficacy in being flexible. (4) The Modification of Goal Adjustment Scale (Wrosch et al., 2003) consists of 10 items measuring goal dis-engagement and goal re-engagement. (5) The Modification of Coping Flexibility and Trauma (Bonanno et al., 2011) consists of 20 items measuring dealing and overcoming trauma.

In treating values as a dynamic structure, Consistency was operationalized as the degree the value structure of the 10 universal values (Schwartz, 1992) motivates personal goals and influences daily actions. In generating items for Consistency, we constructed 14 multiple-fold items that each assessed the10 value domains (see Table 1 for an example). In line with its definition, we constructed six items for Self/Others Ratio that aimed to assess the degree the 10 value domains motivate a person to benefit their *self* and *others* at different levels of functioning (i.e., values, goals, and daily actions). Three out of the six items were candidates for the nominator and three for the denominator of the Self/Others Ratio. Descriptions of value-related items derived from Schwartz’s (1992) value descriptions and were framed as self-related statements that aimed to make values salient in people’s awareness (e.g., “I enjoy life to the fullest” for Hedonism). Placing attention on language clarity and simplicity, we used short positive statements that included a single idea. Some items were reverse-coded.

**TABLE 1:** Example of a 10-fold item assessing the 10 values.

Value domain	Value description
Self-Direction	I decide about which way my life goes.
Stimulation	I am open to discovering new things in life.
Hedonism	I can enjoy life to the fullest.
Achievement	I strive to do my best and succeed in what I do.
Power	I strive to gain resources and influence others.
Security	I want to be safe wherever I am.
Conformity	I follow social rules out of respect for others.
Tradition	I follow ideas of my culture or religion.
Benevolence	I care about my family, friends and other people.
Universalism	I care about all things on the planet.

*Items were rated in response to the stem item: “My most important goals show that.”*

#### Content Validation

Concerning the Flexibility subscale, the process of content validation did not require consulting experts as we relied on established scales that overlapped with our conceptualization of Flexibility. We first reduced the pool of 72 items down to 18, based on two criteria: (i) items were semantically unique and (ii) they could directly answer the generic item stem: “In a situation where I cannot pursue my most meaningful and important goals” (e.g., “I adapt my plans to the new circumstances quite easily”). Regarding items for Consistency and Self/Others Ratio, we identified three individuals with theoretical and practical expertise in values and well-being, who served as content experts. In an online group setting, we received feedback from the three experts who all agreed on retaining eight out of the 14 multiple-fold items in the Consistency subscale and on eliminating six items that were rated as content invalid. All three experts agreed on retaining the six items as candidates for the Self/Others Ratio and on allowing factor analysis to reveal the two most appropriate items for the ratio’s nominator and denominator (Almanasreh et al., 2019). To make sure that our instrument is easy to understand by participants, we worked closely with 25 volunteers from the public who helped us define the questions in a way that evoked intuitive and accurate responses. The sample’s ages ranged from 23 to 82 (*M* = 45.22, *SD* = 11.56, females = 12) and consisted of a variety of nationalities (five White Americans, three White English, nine Germans, six Greeks, one Mexican, and one Mexican–Italian). Participants read the purpose of our investigation and agreed to participate. In one-to-one interviews, we evaluated item clarity and reformulated the items using common expressions. We eliminated one multiple-fold item that participants did not commonly understand. Finally, in an online focus group setting, we discussed item modifications until there were no ambiguities among participants regarding the meaning of the items. Participants agreed that the final questionnaire did not engage them in a strenuous cognitive effort, which added strength to the measuring tool (Barker et al., 2015). The content validation process resulted in 13 items, seven for Consistency and six for Self/Others Ratio. The complete questionnaire is provided in Appendix A.

### Cross-Validation

#### Procedures and Participants

Data collection adhered to the ethical guidelines provided by the University’s Ethics Committee of human subjects. The study did not require ethical approval as it fulfilled the Committee’s criteria for ensuring safe procedures. Prior to entering the study, participants gave their informed consent (Appendix B). Participants who met our inclusive criteria of being a native English speaker and confirming no clinical history of mental health issues received an automatic email link to the online questionnaire. Regarding sample size, although there is no defined minimum sample size for exploratory factor analysis (EFA) (MacCallum et al., 1999), we followed the recommended rule of thumb ratio of 15 participants per one variable, aiming for a large size (Pett et al., 2003; Costello and Osborne, 2005). Accordingly, a sample of 465 participants was deemed sufficient for EFA. As we planned a within-study validation, we aimed to recruit twice as many participants. Participants were recruited via Respondi, an online survey platform, and received CHF 2 in exchange of a 12-min participation, using their own computer. We collected data (*N* = 933) from a national sample in the United Kingdom (age *Mean* = 43.51, *SD* = 13.42, females = 50%) that ranged in age from 18 to 68 years. We stopped data collection when we reached equal number of males and females, distributed equally across three age groups of 18–34, 35–50, and 51–68. When extracting subsets from the whole dataset, we retained equal distribution of males and females across the three age groups. See Appendix C: Table 1 for more details on the samples we extracted for cross-validation purposes.

#### Measures

##### Flexibility

Flexibility items included 18 questions that derived from the scale validation process. Some items were slightly adapted to reflect the conceptualization of Flexibility as ability to re-adjust meaningful and important goals. Items were answered in response to a stem item that suggested a hypothetical scenario in which the most meaningful and important goals became unattainable (e.g., “I think about what else is important to me”).

##### Consistency

Consistency items included seven 10-fold items that assessed: (1) The degree the 10 value domains were integrated as an individual’s value pattern (e.g., Self-Direction: “In an ideal world I decide about which way my life goes”); (2) The degree the value pattern influences daily actions (e.g., Hedonism: “In my daily life I enjoy life to the fullest”); (3) The degree the value pattern remains unfulfilled (reverse-coded) (e.g., Power: “Sometimes I wish I had resources and influence over others”); (4) The degree the value pattern guides personal goals (e.g., Tradition: “My most important goals show that I respect cultural traditions”); (5) The degree the value pattern conflicts with personal goals (Reverse-coded) (e.g., Stimulation: “My personal goals stop me from discovering new things in life”); (6) The degree the value pattern is salient in awareness (e.g., Universalism: “Over the last week, I thought it is important to me that I protect all things on the planet”); and (7) The degree other people’s values influence value integration (e.g., “Seeing other people making their own decisions encourages me to do the same”).

###### Self/Others ratio

Three pairs, two of which contained 10-fold items, aimed to assess Self/Others Ratio at different levels. Items assessed the degree the 10 value domains, goals, and daily actions are perceived to serve *self* and *others*’ interest. The items that would load higher on this factor would form the nominator and denominator of the ratio.

##### Well-Being

Five established well-being measures served as the dependent variables. (1) The SWLS (Diener et al., 1985) consists of five items measuring life satisfaction (e.g., “In most ways my life is close to ideal”). The following three well-being scales were included as research evidence associates them positively with SWLS (King et al., 2006; Steger and Kashdan, 2007). (2) The Multidimensional Existential Meaning Scale (MEMS; George and Park, 2016) involves 15 items that measure comprehension, purpose, and mattering (e.g., “My life makes sense”). (3) The Subjective Happiness (SH; Lyubomirsky and Lepper, 1999) consists of four items measuring subjective judgments of happiness (e.g., “In general, I consider myself a very happy person”). (4) The shortened version of the Psychological Well-Being scale (PWB; Ryff et al., 1995; Dierendonck, 2005) consists of 14-item questionnaire measuring self-acceptance, positive relations with others, autonomy, environmental mastery, purpose in life, and personal growth (e.g., “I am quite good at managing the many responsibilities of my daily life.”). Finally, we included the Perceived Stress (PS; Cohen et al., 1994), that is negatively associated with the SH (Ruiz-Aranda et al., 2014) and the PWB scales (Alleyne et al., 2010). The PS consists of 10 items measuring perception of stress (e.g., “Felt unable to control the important things in life, over the past month”).

Participants rated all measures on a scale from 1 (*not at all* or *strongly disagree*) to 7 (*very much* or *strongly agree*). All well-being scales demonstrated good reliability in all the data subsets. The *alpha* and *omega* coefficients of each scale are reported within the results of each study and some are presented in Appendix C: Table 12.

#### Analytic Strategy

Before collapsing the multiple items into their mean scores, we assessed their reliability by conducting internal consistency tests (Appendix C: Table 2). In addition, we computed the mean scores for our five well-being measures. In all analyses, error rate was 0.05 and *t*-tests were two tailed. Studio *R* (version 1.3.959) was used for the main analyses and SPSS (version 26) was a complimentary software. *R* packages included: corpcor, devtools, polycor, lavaan, simsem, semPlot, psych, boot, GPArotation, lme4, Matrix, expss, lessR, effsize, MeMoBootR, relaimpo, ci.reliability, and stats.

*Study 1* served to examine the factorial structure of the questionnaire that resulted from the content validation process and to identify the items for the nominator and the denominator of the Self/Others Ratio. We administered the 31-item questionnaire on Sample 1 (*n* = 468). First, we ran internal consistency tests on the seven items intended to measure Consistency and on the 18 intended to measure Flexibility and eliminated the items that increased the reliability of each subscale to its maximum (Cronbach, 1951). To ensure that two was an appropriate number of factors to extract, we consulted eigenvalues, scree plot, and Parallel Analysis. Multiple tests confirmed sampling adequacy and item appropriateness for factor analysis. EFA was performed with promax rotation, due to the assumed interrelationship between the hypothesized factors (Costello and Osborne, 2005). In aiming to build an instrument with subscales that each of their items loads high on their latent factor, we eliminated items with factor loadings < 0.70. We also aimed to retain an equal number of items in each subscale as we wished to examine the second-order structure of the instrument in the following study. Finally, we conducted EFA on the six items that intended to assess motivation to benefit *self* and *others* to identify the one with the highest loading for *self* and the one with the highest loading for *others*.

*Study 2* served to test our theoretical assumptions regarding the factorial structure of Psychological Balance and the moderating role of Self/Others Ratio between Psychological Balance and well-being. Will factor analysis confirm that the two subscales intended to assess Consistency and Flexibility load on two distinct factors? Will these factors load on a second-order latent variable that we call Psychological Balance? Are there differences on well-being scores based on participants’ Self/Others Ratio? Does Self/Others Ratio influence the relationship between Psychological Balance and other well-being measures? We administered the items that resulted from *study 1* on Sample 2 (*n* = 465). Confirmatory factor analysis (CFA) with maximum likelihood tested the structure of the reduced questionnaire. Hierarchical confirmatory factor analysis (HCFA) (Messick, 1989, 1995) tested the structure of our theoretical model. Internal consistency of the resulting subscales and the five well-being variables was assessed by calculating both their *alpha* and *omega* coefficients (Revelle and Zinbarg, 2009; Crutzen and Peters, 2017; McNeish, 2018; Dunn et al., 2019). Confidence intervals (CI) in relation to *alpha* and *omega* values refer to 95% CI and when reported in the text are included in square brackets. As a means to scale validation, we conducted Pearson’s correlations and regression analyses to examine the relationship of each subscale to the five depended variables. Relative importance analyses demonstrated the amount of variance in the well-being variables explained by each factor of Psychological Balance (Johnson and LeBreton, 2004; Tonidandel and LeBreton, 2011). To confirm the two most reliable items for the nominator and the denominator of Self/Others Ratio, we conducted CFA on the six items intended to assess this variable. We dummy coded Self/Others Ratio (e.g., 0 = within the range and 1 = outside the range) using the range that yielded relatively even size groups. Then, we performed *t*-tests to compare differences in Consistency and Flexibility and in the five well-being variables between participants with a ratio coded as 0 and 1. Performing moderation analyses using multiple linear regression models investigated the influence of Self/Others Ratio on the relationship between Consistency, Flexibility, and the five well-being variables. Finally, gender differences were examined using *t*-tests and differences between the means of the three age groups were explored with multiple comparisons in analysis of variance.

In *study 3*, we utilized the whole sample (*N* = 933) to further explore gender and age differences and tested measurement invariance across gender and age by administering the emerging measures on five subsets of the data. Will the instrument display psychometric properties when administered to different age and gender groups? We conducted multiple group confirmatory factory analysis (MGCFA; Milfont and Fischer, 2010) on Samples 3 (*n* = 466, 100% males) and 4 (*n* = 467, 100% females) representing gender and on Samples 5 (*n* = 311, 18–34 years of age, 50% females), 6 (*n* = 311, 35–50 years of age, 50% females), and 7 (*n* = 311, 51–68 years of age, 50% females) representing age. In addition, we tested measurement invariance across the two groups of Self/Others Ratio (0.95–1.05 range). We explored differences in well-being variables by comparing mean differences between Self/Others Ratio groups, based on three ratio ranges. Multiple linear regression investigated the interaction effect of the Self/Others Ratio on the relationship between the two sub-constructs of Psychological Balance and the five well-being variables. The effect size *f*^2^ of the moderation (Aiken et al., 1991) in *study 2* informed *a priori* power analyses for the moderation analyses.

## Results

### Study 1

#### Factorial Structure

**FIGURE 3:**
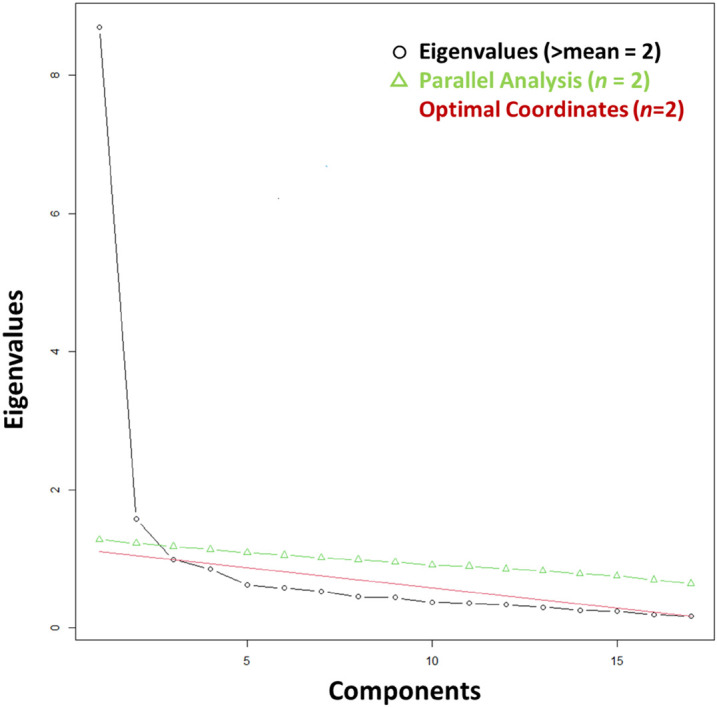
Study 1: Scree plot assessed appropriate number of factors for exploratory factor analysis.

Internal consistency tests on the pool of items that aimed to assess Consistency and Flexibility, respectively, indicated the eight items that their elimination improved internal consistency within each subscale (Appendix C: Table 3). To examine the factorial structure of the remaining 17 items, we conducted EFA. As expected, two factors had eigenvalues greater than 1 and the scree plot and parallel analysis both justified retaining two factors (Figure 3). An initial KMO test verified great sample adequacy for the analysis as the Kaiser–Meyer–Olkin (KMO) value was 0.94 and all the individual items were > 0.90 (Field et al., 2012). EFA procedures showed that eight items had factor loadings < 0.70 and Flex_13 did not load on any of the two factors. We repeated an EFA on the eight remaining items and eliminated Flex_3 that had a factor loading of 0.68 and Flex_12 that had a factor loading of 0.67 (Table 2). The third EFA showed that the six remaining items had factor loadings > 0.70 (Appendix C: Table 4). A good determinant of 0.038 indicated that there was no multicollinearity in the correlation matrix of the remaining items as this was greater than 0.00001 (Appendix C: Table 5). We re-ran tests to examine sampling adequacy. The Bartlett’s test of sphericity had a significant value (χ*^2^* 15) = 1512.133, *p* < 0.001, confirming that correlations between variables were significantly different from zero. The KMO of the correlation matrix was 0.79 and KMO values for individual items were all very good (0.74–0.88), showing sampling adequacy for factor analysis.

**TABLE 2:** Study 1: Pattern matrix from exploratory factor analysis.

		Pattern coefficients		
		Factor 1	Factor 2	*h* ^2^
C1^a^	In an ideal world.		0.61	0.44
C2	In my daily life.		0.94	0.75
C4	My most important goals show that.		0.90	0.66
C6	It is very important (to me) that.		0.71	0.45
F1^a^	I adapt my goals to the new circumstances quite easily.	0.61		0.51
F2^a^	I remind myself that other things in life are just as important.	0.49		0.53
F3^b^	After a serious drawback, I soon turn to new goals.	0.75		0.54
F5^a^	I have many possible ways of pursuing my goals in any given situation.	0.59		0.51
F6^a^	I am willing to consider alternative ways to pursue my goals.	0.55		0.47
F8	I seek other meaningful goals.	0.93		0.67
F9	I start working on other new goals.	0.98		0.72
F11^a^	I think about what else is important to me.	0.64		0.53
F12^b^	I consider what other goals I could achieve under the circumstances.	0.83		0.66
F13^a^	I think about what exactly I really want.			0.34
F14^a^	I direct my efforts at what is still possible.	0.53		0.50
F15	I re-define my goals.	0.88		0.62
F16^a^	I reflect on the meaning of the event.	0.44		0.37

*n = 468. C, consistency; F, flexibility; h^2^, communalities. ^a^Items reduced after the first EFA analysis. ^b^Items reduced after second EFA analysis.*

#### Reliability

The new scale demonstrated a very good reliability. In Psychological Balance scale, Cronbach’s *alpha* and Revelles’ *omega* were, respectively: α = 0.84 [0.82, 0.86], ω = 0.91 [0.84, 0.88]; in Consistency: α = 0.83 [0.80, 0.86], ω = 0.84 [0.80, 0.86]; and in Flexibility: α = 0.88 [0.87, 0.90], ω = 0.89 [0.87, 0.91]. Internal consistency in the well-being variables was also good.

#### Self/Others Ratio

Exploratory factor analysis revealed the most reliable items for the nominator and denominator of the ratio (Appendix C: Table 6). We computed the mean ratio of SOR3 (i.e., “Degree of motivation by the 10 values to benefit the self”) to SOR4 (i.e., “Degree of motivation by the 10 values to benefit others”), *M* = 1.09, *SD* = 0.31.

### Study 2

In Sample 2 (*n* = 465), we subjected the six items that resulted from the last EFA analysis in *study 1* to a CFA, aiming to replicate their factor structure. Two factors were specified, and maximum-likelihood estimation was used with promax rotation. The model had a very good fit, χ*^2^*(8) = 20.299, *p* = 0.009, confirmatory fit index (CFI) = 0.991, Tucker–Lewis index (TLI) = 0.983, root-mean-square error of approximation (RMSEA) = 0.057, and standardized root-mean-square residual (SRMR) = 0.030. The two factors had a correlation of *r* = 0.58. CFA confirmed the results of the EFA in *study 1* (Appendix C: Table 7), and we re-coded the items of the final scale of Psychological Balance (Table 3). We report the new six-item scale in full detail in Appendix D. The three items indicating factor 1 suggested that the Flexibility subscale represented cognitive ability to re-adjust the most meaningful and important goals in the face of challenge. The three items indicating factor 2 suggested that the Consistency subscale represented the degree the 10 value domains motivate personal goals and manifest in daily actions.

**TABLE 3:** Study 2: Pattern matrix from confirmatory factor analysis.

		Pattern coefficients	
		Flexibility	Consistency
F1	I start working on new goals.	0.91	
F2	I seek other meaningful goals.	0.86	
F3	I re-define my goals.	0.70	
C1	My most important goals show that…		0.71
C2	In my daily life…		0.98
C3	It is very important (to me) that…		0.76

*n = 465. C, consistency; F, flexibility.*

#### Reliability

Internal consistency results were very good also in Sample 2, as the composite score of Psychological Balance displayed a Cronbach’s *alpha* coefficient of 0.85 [0.83, 0.87] and Revell’s *omega* coefficient of 0.92 [0.85, 0.89]. Consistency displayed α = 0.86 [0.84, 0.88] and ω = 0.87 [0.85, 0.89], and Flexibility displayed α = 0.85 [0.83, 0.88] and ω = 0.86 [0.84, 0.88]. Reliability of the established measures was also good. MEMS: α = 0.95 [0.94, 0.95], ω = 0.97 [0.94, 0.95]; SWLS: α = 0.91 [0.90, 0.92], ω = 0.93 [0.90, 0.92]; SH: α = 0.68 [0.63, 0.72], ω = 0.34 [0.74, 0.81]; PWB: α = 0.84 [0.82, 0.86], ω = 0.88 [0.80, 0.85]; and PS: α = 0.88 [0.87, 0.90], ω = 0.93 [0.87, 0.90].

#### Validity

**FIGURE 4:**
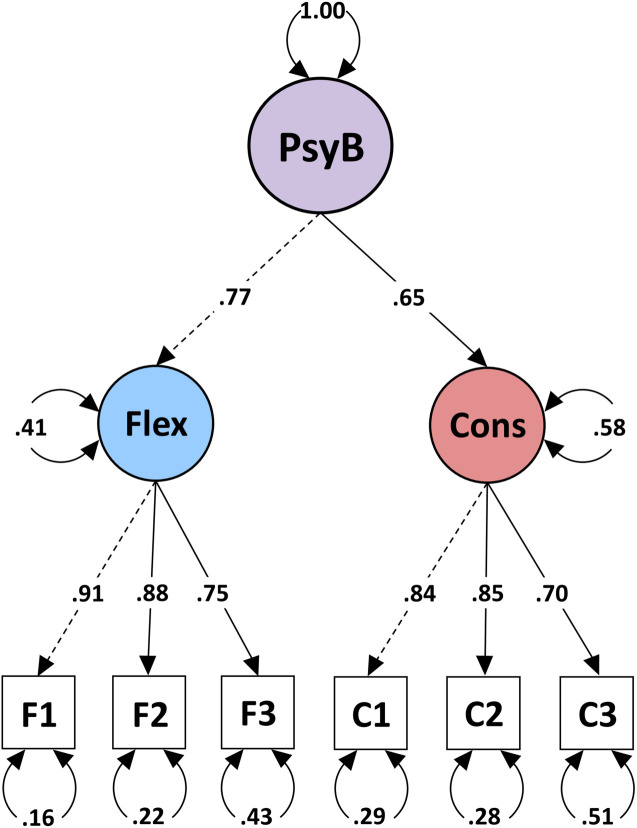
Study 2: High-order structure of Psychological Balance.

Toward the validation of our theoretical model, we examined the second-order structure of the six items by performing HCFA. We specified a model with Psychological Balance as a second-order factor indicated by Consistency and Flexibility, each measured by three observed variables. The model had a very good fit, χ*^2^*(8) = 20.299, *p* = 0.009, CFI = 0.991, TLI = 0.983, RMSEA = 0.057, and SRMR = 0.030 (Figure 4) (for full results, see Appendix C: Table 8). A nested model with a single latent factor did not converge (Hu and Bentler, 1999; Kline, 2010).

In testing the scale’s convergence and discriminant validity, we investigated the relationship of the two emerging factors with the five theoretically relevant variables using correlation, regression, and relative importance analyses (Table 4). Results indicated that: (a) Flexibility and Consistency correlated positively with MEMS, SWLS, SH, and PWB and negatively with PS; (b) both factors predicted a significant amount of variance in theoretically relevant well-being variables; and (c) both factors explained a significant amount of variance in each of the well-being variables, as indicated by their relative weights. The exception was Flexibility that was not a significant predictor of PWB and PS, when Consistency was the second predictor in the model. For example, Consistency and Flexibility were both significant predictors in MEMS and together accounted for 33% of its variance; 67% of this amount was attributed to Consistency and 33% to Flexibility. These results provided strong evidence toward the validity of the new scale. HCFA validated the second-order structure of Psychological Balance.

**TABLE 4:** Study 2: Relationships of consistency and flexibility with theoretically related variables.

	*r*	*β*	*p*	RW[CI]	R-RW[%]
***MEMS***					
Consistency	0.55**	0.68	<0.001	0.234 [0.162–0.303]	66.69
Flexibility	0.43**	0.24	<0.001	0.117 [0.067–0.174]	33.30
Model R^2^	/	0.33	<0.001		
***Satisfaction with Life***					
Consistency	0.39**	0.41	<0.001	0.113 [0.065–0.171]	49.37
Flexibility	0.41**	0.35	<0.001	0.069 [0.053–0.177]	50.63
Model *R*^2^	/	0.22	<0.001		
***Subjective Happiness***					
Consistency	0.41**	0.33	<0.001	0.114 [0.069–0.175]	45.90
Flexibility	0.45**	0.33	<0.001	0.151 [0.072–0.200]	54.09
Model *R*^2^	/	0.25	<0.001		
***Psych. Well-Being***					
Consistency	0.40**	0.38	<0.001	0.164 [0.108–0.230]	76.69
Flexibility	0.24**	0.05	0.186	0.050 [0.019–0.091]	23.31
Model *R*^2^	/	0.16	<0.001		
***Perceived Stress***					
Consistency	–0.15**	–0.17	0.026	0.018 [0.009–0.056]	60.23
Flexibility	–0.14*	–0.08	0.135	0.012 [0.001–0.045]	39.76
Model R^2^	/	0.03	<0.001		

MEMS, Multidimensional Existential Meaning Scale; r, correlation coefficient; β, regression coefficient; p, significance value for beta coefficient; RW[CI], relative weight and associated confidence interval; R-RW, relative weight rescaled as a percentage of the total model variance. *p <0.05. **p < 0.01.

#### Age and Gender

There were no statistically significant gender- nor age-related differences in Consistency and Flexibility in Sample 2.

#### Self/Others Ratio

Confirmatory factor analysis with maximum likelihood and without rotation replicated SOR3 (0.71) and SOR4 (0.73) as the items with the highest factor loadings (Appendix C: Table 9). The mean of Self/Others Ratio was *M* = 1.09 and it ranged from a minimum of 0.39 to a maximum of 7. A binary dummy coded variable based on a ratio range of 0.95–1.05 (i.e., 0 = outside the range, *n* = 262 and 1 = within range, *n* = 203) helped to investigate Self/Others Ratio group differences in Consistency, Flexibility, and the five well-being variables. Results of *t*-tests showed that participants with a Self/Others Ratio within the range of 0.95–1.05 (1) (*M* = 5.37, *SD* = 0.83) reported significantly higher levels in Consistency than those with a Self/Others Ratio outside the range of 0.95–1.05 (0) (*M* = 5.07, *SD* = 0.88), *t*(463) = 3.76 *p* = 0.001. Also, participants with 1 (*M* = 4.86, *SD* = 1.22) reported significantly higher levels in Flexibility than those with 0 (*M* = 4.43, *SD* = 1.14). There were no statistically significant differences in the means of PWS and PS between the two ratio groups. Appendix C: Table 10 reports further results. Furthermore, we created a binary-coded variable based on a ratio range of 0.80–1.20 to test our hypothesis that beyond a critical Self/Others Ratio, the relationship between Consistency and Flexibility and well-being bifurcates. Multiple linear regression revealed that after controlling for age, there was a statistically significant interaction effect of Self/Others Ratio on the relationship between Flexibility and the MEMS, with a medium to large effect size *f*^2^ = 0.25. The ratio also moderated the relationship between Consistency and the SH scale, with a medium to large effect size *f*^2^ = 0.26 (Appendix C: Table 11). These results indicate that in *Sample 2*, the Self/Others Ratio of within and outside the range of 0.80–1.20 was a positive moderator of the relationship between Consistency and happiness and Flexibility and meaning.

### Study 3

#### Measurement Invariance

**FIGURE 5:**
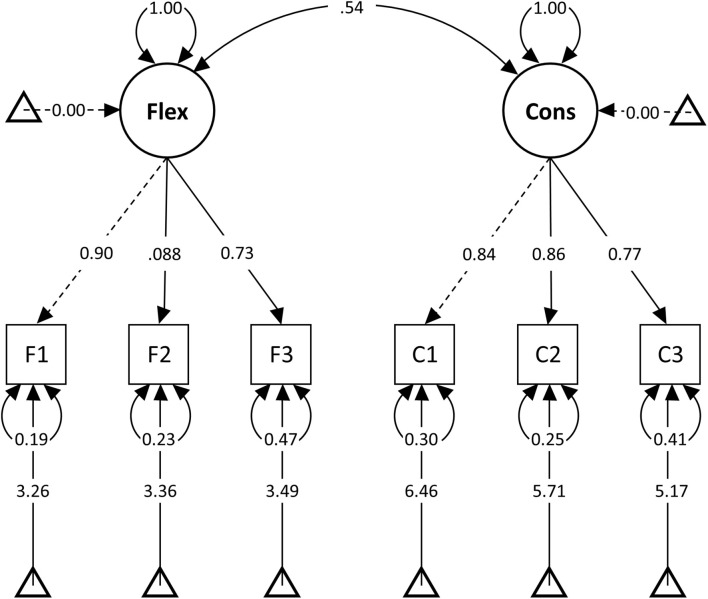
Study 3: Measurement model for testing measurement invariance.

The first objective of *Study 3* was to test measurement invariance across gender and age. As the new instrument aimed to assess latent psychological constructs, we tested whether participants ascribed the same meaning to the questions. All measures demonstrated good Cronbach’s *alpha* and Revelle’s *omega* values across all the data subsets (Appendix C: Table 12). MGCFA determined *scalar invariance* across gender and age by increasingly restricting free parameters in hierarchical nested models (Steenkamp and Baumgartner, 1998; Lee, 2018). First, we built a measurement model using the whole dataset by specifying the relationships between the second-order factor of Psychological Balance and its two latent factors of Flexibility and Consistency, indicated by three observed variables each. All items loaded strongly on their latent variable, variances were all positive, and *r*^2^ were less than 1, whereas goodness-fit-indices showed that the model fitted the data well: χ*^2^*(8) = 27.999, *p* < 0.001, CFI = 0.994, TLI = 0.988, RMSEA = 0.052, and SRMR = 0.021 (Bollen, 1989; Figure 5). A nested model comparison ensured that the overall model performed equally well across the two gender groups, allowing us to proceed with the analyses (Schermelleh-Engel et al., 2003; Appendix C: Tables 13, 14). Then we built a series of nested models and compared their CFI indices (Cheung and Rensvold, 2002). Testing *configural invariance* examined whether the overall factorial structure of our scale was a good fit across males and females when factor loadings and intercepts were free to vary. Next, in testing *metric invariance*, we constrained the factor loadings to be equal across gender, whereas intercepts were free to vary. A good model fit with a CFI difference of 0.004 indicated equivalent factor loadings across gender groups. In the third step of *scalar invariance*, we added the constraints of equivalence among intercepts. The model continued to have a good fit and a CFI difference of 0.003 from the previous model, confirming that any statistical differences in group means were not due to gender differences in scale properties. To test measurement invariance across the three age groups, we repeated the above analyses using Samples, 5, 6, and 7. A nested group comparison ensured that the overall model performed well across the three age groups, which allowed us to proceed with the multiple group analyses (Appendix C: Tables 15–17). Then, we followed the three sequential steps by first adding constraints to the configural model. Overall CFI differences less than 0.01 between configural, metric, and scalar models confirmed measurement invariance across the three age groups (Table 5).

**TABLE 5:** Study 3: Comparison of fit indices across gender, age, and Self/Others Ratio groups.

	*χ* ^2^	*df*	CFI	RMSEA [CI]	SRMR	CFI.delta	RMSEA.delta
***Males and females (Samples 3 and 4)***							
Configural	37.55	16	0.993	0.054 [0.031–0.076]	0.024	NA	NA
Metric	54.48	21	0.989	0.058 [0.040–0.078]	0.091	0.004	0.004
Scalar	49.42	24	0.992	0.048 [0.028–0.067]	0.028	0.003	0.010
***Age groups: 18–34, 35–51, 52–68 (Samples 5, 6, and 7)***							
Configural	41.85	24	0.994	0.049 [0.022–0.073]	0.024	NA	NA
Metric	50.39	32	0.994	0.043 [0.017–0.065]	0.031	0.000	0.006
Scalar	79.50	40	0.987	0.056 [0.038–0.074]	0.038	0.007	0.013
***Self/Others Ratio (0.95–1.05)***							
Configural	36.97	16	0.933	0.053[0.031–0.076]	0.025	NA	NA
Metric	42.55	20	0.933	0.049[0.028–0.070]	0.033	0.000	0.004
Scalar	45.19	24	0.933	0.044[0.023–0.063]	0.033	0.000	0.005

*df, degrees of freedom; CFI, comparative fit index; RMSEA, root mean square error of approximation; CI, confidence interval; SRMR, standardized root mean square residual; CFI.delta, consecutive model differences in CFI; RMSEA.delta, consecutive model differences in RMSEA.*

#### Age and Gender

There were no statistically significant age- nor gender-related differences in Consistency and Flexibility in the whole dataset (*N* = 933). We reported detailed results regarding our explorations in age- and gender-related differences in Appendix C: Tables 18, 19.

#### Self/Others Ratio

Dummy coding Self/Others Ratio based on three ranges (i.e., 0.95–1.05, 0.90–1.10, and 0.80–1.20) allowed us to examine differences in Psychological Balance scale and the five well-being variables between participants “within the range” and those “outside the range.” To maximize power when conducting two-tailed independent *t*-tests, we aimed to use equal size groups (Kim and Park, 2019) of participants with a Self/Others Ratio “within the range” and “outside the range.” Hence, in each range, the sample size was driven by the smaller size group (Appendix C: Table 20). Given our inclusion criterion of not having reported any mental health issues restricted the exploration of wider ranges (i.e., 0.50–1.50) as only a few participants belonged to the group outside such ranges. In ensuring that participants in both groups perceived the Consistency and Flexibility subscales in a similar way, we tested measurement invariance across the Self/Others Ratio groups. Since scalar invariance was met (see Table 5), we continued with further analyses. A series of *t*-tests revealed statistically significant differences between the two Self/Others Ratio groups in Consistency, Flexibility, as well as in SWLS, MEMS, and SH. The ratio range 0.90–1.10 revealed statistically significant differences between the two groups also in PWB and PS (Appendix C: Table 21).

**FIGURE 6:**
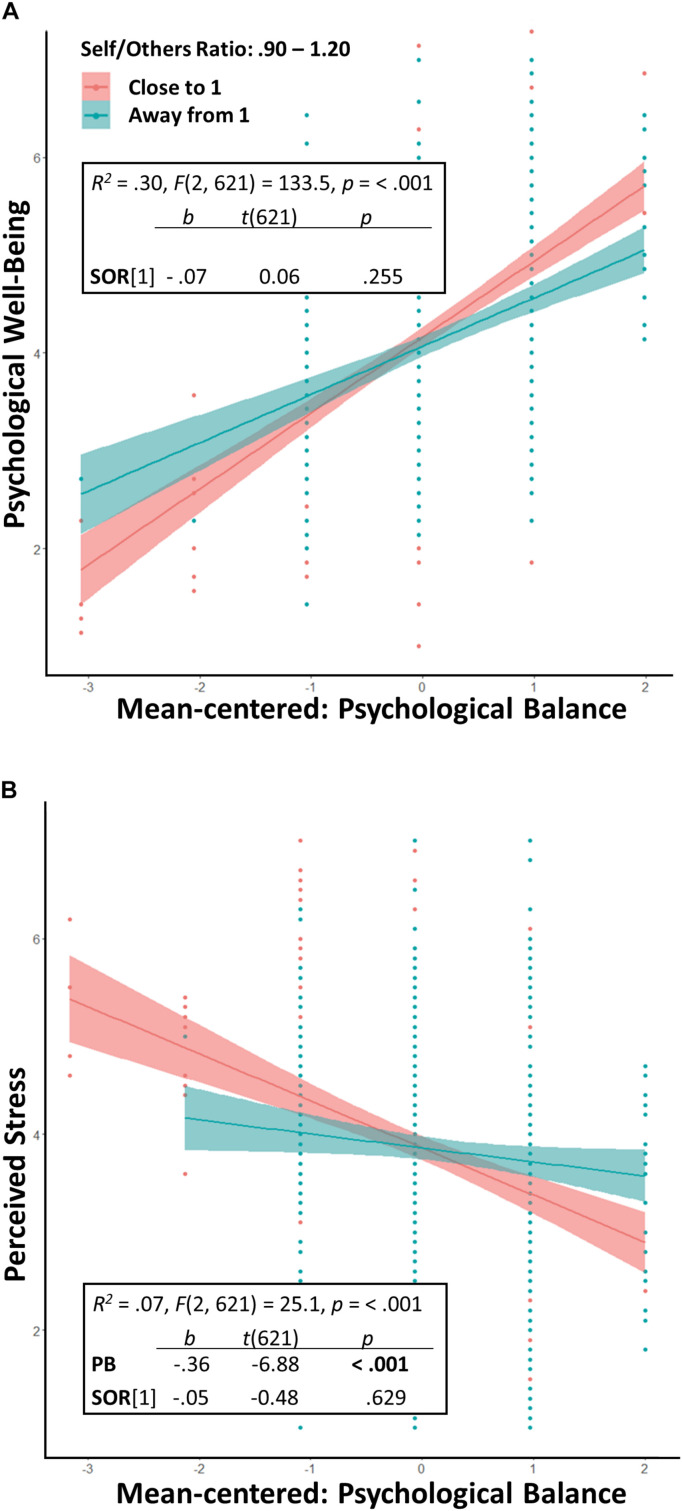
Study 3: **(A)** Self/Others Ratio as a positive moderator in the relationship between Psychological Balance and Psychological Well-Being. **(B)** Self/Others Ratio as a negative moderator in the relationship between Psychological Balance and Perceived Stress.

We performed moderation analyses using the three ranges of Self/Others Ratio as the moderator variable. *A priori* power analysis using G^∗^ Power 3 (Faul et al., 2007) revealed that a minimum sample of 54 participants would suffice for detecting a medium to a large interaction effect *f^2^* = 0.25 of Self/Others Ratio on the relationship between Flexibility and well-being, at a high power of 0.90 at an *alpha* level of 0.05. Results showed an interaction effect of the ratio range 0.95–1.05 in the relationship between the Psychological Balance scale and PWB and with the PS. Table 6 shows the statistically significant results of moderation analyses in detail. When Consistency was the predictor variable of PWB, its interaction with Self/Others Ratio was statistically significant. When Flexibility was the predictor variable, its interaction with Self/Others ratio was also statistically significant. Regarding PS, when Consistency was its main predictor, the interaction between Consistency and Self/Others Ratio was not statistically significant, whereas, when Flexibility was the main predictor of PS, its interaction with Self/Others Ratio was statistically significant. These results identify Self/Others Ratio (0 = outside 0.95–1.05 and 1 = within 0.95–1.05) as a positive moderator of the relationship between Psychological Balance and PWB and as a negative moderator between Psychological Balance and PS (Figures 6A,B).

**TABLE 6:** Study 3: Results of moderation analyses with Self/Others Ratio as the moderator.

	Psychological Well-Being			Perceived stress		
	*β*	CI	*p*	*β*	CI	*p*
	***Consistency as main predictor***					
Model fit	*F* (4,814) = 77.69			*F* (4,814) = 24.26		
Consistency	0.59	[0.50 to 0.67]	0.001	–0.45	[–0.59 to 0.31]	0.001
SOR [1]	–0.14	[–0.24 to –0.03]	0.009	0.02	[–0.16 to 0.19]	0.815
Age Group	0.10	[0.04 to 0.16]	0.002	–0.27	[–0.038 to –0.17]	0.001
Interaction	–0.13	[–0.25 to –0.001]	0.029	0.06	[–0.14 to 0.26]	0.541
	***Flexibility as main predictor***					
Model fit	*F* (4,814) = 651.08			*F* (4,814) = 21.19		
Flexibility	0.38	[0.32 to 0.45]	0.001	–0.36	[–0.46 to –0.25]	0.001
SOR [1]	0.01	[–0.09 to 0.12]	0.831	–0.10	[–0.27 to 0.07]	0.234
Age Group	0.10	[0.03 to 0.16]	0.004	–0.27	[–0.37 to 0.17]	0.001
Interaction	–0.11	[–0.21 to –0.002]	0.019	0.18	[0.03 to 0.33]	0.016

*n = 820. β, regression coefficient; F, F-statistic; CI, Confidence Interval; SOR, Self/Others Ratio (0.95–1.05).*

#### Validity

**FIGURE 7:**
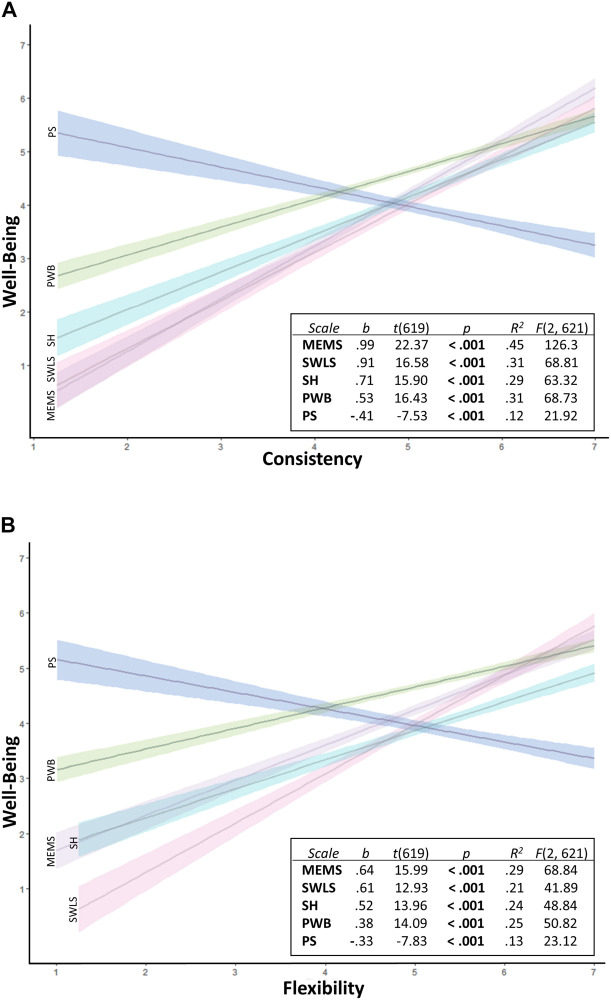
Study 3: **(A)** Replicated regression analyses confirming convergence and divergence validity of the Consistency subscale. **(B)** Replicated regression analysis confirming convergence and divergence validity of the Flexibility. MEMS, Multidimensional Existential Meaning Scale; SWLS, Satisfaction with Life Scale; SH, Subjective Happiness; PWB, Psychological Well-Being; PS, Perceived Stress.

Using the sample extracted based on the 0.90–1.10 Self/Others Ratio range (*n* = 618), we replicated results regarding the associations of Consistency and Flexibility with the five theoretically relevant well-being variables (Figures 7A,B).

Altogether, our instruments for Consistency and Flexibility demonstrated good reliability and validity. Meeting scalar invariance provided further evidence for the robustness of the new measure. As the meaning of the measure was equivalent across gender and age, we can safely compare group differences in Flexibility and Consistency as well as in the Psychological Balance scale. We identified a Self/Other Ratio range that moderates the relationship between Psychological Balance and theoretically relevant well-being variables. Due to some insignificant differences across samples, we refrain from drawing any conclusions regarding age and gender variation in Consistency and Flexibility.

## Discussion

In the present work, we proposed a multi-dimensional tool that aided the empirical investigation of a novel theoretical model. We addressed the following: (a) Development and validation of the scale and the factorial structure of Psychological Balance, comprising Consistency and Flexibility; (b) Development and validation of a measure for Self/Others Ratio, a second-order factor that influences Psychological Balance and moderates its relationship to well-being; and (c) Associations between Consistency, Flexibility, and Self/Others Ratio with well-being variables, as a means to instrument validation. As expected, participants with higher scores in Consistency and Flexibility compared to those with lower scores, and participants with a Self/Others Ratio range close to 1 compared to those with a range away from 1, reported a happier, more satisfying and meaningful life and overall well-being and lower levels of stress.

On the one hand, the Consistency subscale assessed the degree the 10 value domains (Schwartz, 1992) consciously motivated participants and influenced their goals and daily actions. The positive relationship of Consistency with the SWLS and SH converges with previous research evidence that associates life satisfaction with fulfilling as many life domains as possible (e.g., Palys and Little, 1983) and another source that indicates that people try to satisfy all the different things they value most, at least at a moderate level (Reiss, 2004). The Consistency subscale explained a significant amount of variance in the MEMS, indicating that the 10 value domains provide a sense of coherence of the self in relation to the external world when they inform personal goals and manifest in daily behavior (George and Park, 2016). On the other hand, the Flexibility subscale assessed the level of ability to re-define meaningful and important goals in the face of challenge. This converges with the idea that people seek meaning in different domains to the one in which meaning is threatened (Heine et al., 2006) and also indicates fluctuations in the individual’s value pattern (see Figure 1). Our findings of a positive relationship between the Flexibility subscale and the SWLS and SH are in line with previous findings that show that flexible cognitive re-adjustments can restore emotional balance (Higgins, 1987; Higgins and Kruglanski, 2000). The ability to modify new information to fit into what a person already knows and restructure what they already know to accommodate new information determines successful adaptation (Brandtstädter and Greve, 1994). Regarding Self/Others Ratio, results demonstrated that integrating the two mental contexts at a similar level is associated with an overall higher level of well-being compared to prioritizing self over others and vice versa. This is in line with previous findings showing that caring for others serves to enhance the self as it fosters coherence within the self-concept and in a person’s relationship with the external world (Park, 2010). In addition, moderation analyses showed that Self/Others Ratio influenced the relationship of both sub-constructs of the Psychological Balance scale to PWB, as well as the relationship of the Flexibility subscale to PS. Altogether, this primary empirical investigation validated our theoretical conceptualization of Psychological Balance.

### Limitations

Although the new instruments met scalar measurement invariance, our sample was not culturally diverse. In addition, participants that engage in online studies may not be strictly representative of a population and our exclusion criteria of not having previously reported mental health issues paused a limitation on identifying a critical Self/Others Ratio. Despite having used a large sample to test our theoretical assumptions, the data come from a cross-sectional study and longitudinal data are needed to further test the fluctuations of the dynamic constructs we introduce.

### Future Research

Future research may test measurement invariance across cultures. Identifying differences in individual value patterns may also give an insight into value profile variance across cultures. Flexibility may represent different meanings across cultures as possibly different cultures deal differently with having to adapt their goals to a situational challenge. Identifying the critical Self/Others Ratio beyond which individuals are impacted adversely by change and present with symptoms of psychopathology requires further investigations within a clinical setting. Another area that can be addressed in future work is to investigate the individual relationships of Consistency, Flexibility, and Self/Others Ratio to the three sub-constructs of the MEMS (e.g., coherence, purpose, and mattering) as well as to the six sub-constructs of PWB (e.g., autonomy, environmental mastery, purpose in life, positive relations with others, and self-acceptance). Longitudinal studies may assess the level of stability and fluctuation of Psychological Balance across time. Identifying temporal and situational contents that may influence this multidimensional construct may aid its investigation and understanding. Experimental research could manipulate value salience to test the effect on Consistency and Flexibility and overall well-being.

## Conclusion

The present work provides validation of a novel theoretical development concerning Psychological Balance, a dynamic state characterized by relatively stable characteristics that can adapt to change. Accordingly, evidence indicates that the integration of the 10 value domains, which constitute a universal value structure, as ideals that inform personal goals and influence behavior, as well as ability to define and pursue new meaningful goals when life events get in the way of a person’s plans, contribute to Psychological Balance. A critical ratio of motivation to benefit personal interest and the interests of other people influences a person’s psychological stability and overall well-being. In conclusion, people with a strong Psychological Balance are likely to feel happy and experience high levels of meaning and life satisfaction and low levels of stress. The proposed theory and instruments may aid future research and contribute to understanding the psychological antecedents of well-being. The Psychological Balance scale may provide a tool for assessing cognitive and behavioral aspects of functioning. The multidimensionality of the new instrument accommodates the investigation of the construct’s fluctuations across time and carries the potential of obtaining an insight into different levels of functioning. Gaining an insight into the problematic areas may form the basis of effective interventions for improving well-being.

## Data Availability Statement

The raw data supporting the conclusions of this article will be made available by the authors, without undue reservation.

## Ethics Statement

The studies involving human participants were reviewed and approved by the Ethics Committee of University of Zurich on human subjects. The participants provided their written informed consent to participate in this study.

## Author Contributions

AB and MM contributed to the conception and design of the study. AB collected and organized the data and wrote the first draft of the manuscript. AB and AH performed the statistical analysis and analyzed the data. All the authors contributed to manuscript revisions and read and approved the submitted version.

## Conflict of Interest

The authors declare that the research was conducted in the absence of any commercial or financial relationships that could be construed as a potential conflict of interest.

## Publisher’s Note

All claims expressed in this article are solely those of the authors and do not necessarily represent those of their affiliated organizations, or those of the publisher, the editors and the reviewers. Any product that may be evaluated in this article, or claim that may be made by its manufacturer, is not guaranteed or endorsed by the publisher.
