# Supplementary material for: Psychological Balance Scale: Validation Studies of an Integrative Measure of Well-Being
Source: Front Psychol. 2021 Sep 16;12:727737. doi: 10.3389/fpsyg.2021.727737 (PMC8483246; doi:10.3389/fpsyg.2021.727737)
Supplement: Supplementary file 1 [file Table_1.docx]

Appendix A

# **31- ITEM QUESTIONNAIRE**

**Instructions**

Please, answer the following questions intuitively. There are no right or wrong answers. Some questions may sound similar. Please, answer them without worrying about your previous responses.

## Consistency

Values refer to ideals, which may motivate and guide people in life. Values typically represent what is most important to people. We are using ten sentences that describe ten social values to help us understand what you value most in life.

*Degree values are part of the ‘value system’*

**C1. To what degree do the following statements describe you?**

*Not at all* *1 2 3 4 5 6 7 Very much*

**In an ideal world…**

1. I decide about which way my life goes. *(Self-direction)*
2. I discover new things in life. *(Stimulation)*
3. I enjoy life to the fullest. *(Hedonism)*
4. I am successful in everything I do. *(Achievement)*
5. I have resources and influence over others. *(Power)*
6. I feel safe wherever I am. *(Security)*
7. I respect other people and follow social rules*. (Conformity)*
8. I accept and follow the ideas of my culture or religion. *(Tradition)*
9. I care about my family, friends and others around me. *(Benevolence)*
10. I care about all things on the planet. *(Universalism)*

*Degree daily actions express personal values*

**C2. To what extent do you agree with the following statements?**

*Strongly disagree* *1 2 3 4 5 6 7 Strongly agree*

**In my daily life...**

1. I decide about what I do.
2. I can discover new things.
3. I enjoy the day to the fullest.
4. I give my best in what I do and strive to succeed.
5. I strive to gain resources and influence others.
6. I make sure I am safe wherever I am.
7. I follow social rules out of respect for other people.
8. I follow the traditions of my culture.
9. I show that I care about my family, friends and others around me.
10. I show that I care about all things on the planet.

*Degree personal values remain unexpressed (R)*

**C3. To what extent do you agree with the following statements?**

*Strongly disagree* *1 2 3 4 5 6 7 Strongly agree*

**Sometimes I wish I could:**

1. Decide more about the way my life goes.
2. Discover more new things in life.
3. Enjoy life more.
4. Try more to be successful in what I do.
5. Have more resources and influence over other people.
6. Feel more safe in my environment and in myself.
7. Follow social rules more.
8. Follow the traditions of my culture more.
9. Care more about my family, friends and others around me.
10. Care more about all things on the planet.

*Degree most important personal values guide important goals*

**C4. Thinking of your most important personal goals altogether, to what extent do you agree with the following statements?**

*Strongly disagree* *1 2 3 4 5 6 7 Strongly agree*

**My most important goals show that:**

1. I decide about which way my life goes.
2. I am open to discovering new things in life.
3. I can enjoy life to the fullest.
4. I strive to do my best and succeed in what I do.
5. I strive to gain resources and influence others.
6. I want to be safe wherever I am.
7. I can follow social rules out of respect for others.
8. I can follow ideas of my culture or religion.
9. I care about my family, friends and other people.
10. I care about all things on the planet.

*Degree personal goals conflict with values (R)*

**C5. Thinking of your personal goals, to what extent do you agree with the following statements?**

*Strongly disagree* *1 2 3 4 5 6 7 Strongly agree* **Some of my goals stop me from:**

1. Deciding which way my life goes.
2. Discovering new things in life.
3. Enjoying life to the fullest.
4. Being the best I can be.
5. Having resources and influencing other people.
6. Feeling safe wherever I am.
7. Following social rules and show respect for others.
8. Following the traditions of my culture.
9. Caring about my family, friends and people around me.
10. Caring about all things on the planet.

*Degree of value salience*

**C6. Over the last week, how often did you think in this (or a similar) way?**

*Never* *1 2 3 4 5 6 7 Very often*

**It is very important (to me) that:**

1. I make my own decisions about my life.
2. I can discover new things in life.
3. I enjoy life to the fullest.
4. I give my best in what I do and succeed.
5. I have resources and can influence others.
6. I feel safe in my environment and in myself.
7. I show respect to others by following social rules.
8. I follow the traditions of my culture.
9. I care about my family, friends and others around me.
10. I protect all things on earth.

*Degree values endorsed by others contribute to value integration*

**C7. To what extent do you agree with the following statements?**

*Strongly disagree* *1 2 3 4 5 6 7 Strongly agree*

**Seeing other people** *(complete with each of the following 10 statements)* **encourages me to do the same.**

1. Making their own decisions about their life...
2. Discovering new things in life...
3. Enjoying life to the fullest...
4. Striving to be the best they can be...
5. Having resources and influencing other people...
6. Being safe in their environment and in themselves...
7. Following social rules out of respect for others...
8. Respecting and following the traditions of their culture...
9. Caring about their family, friends and others’ around them...
10. Caring about all things on the planet...

## Self and Others

*Degree daily actions are perceived to serve self interest.*

**SOR 1. Thinking of your daily actions, to what extent do you agree with the following statement?**

My daily actions help me benefit myself

*Not at all 1 2 3 4 5 6 7 Very much*

*Degree daily actions are perceived to serve others interest.*

**SOR 2. Thinking of your daily actions, to what extent do you agree with the following statement?**

My daily actions help me benefit other people:

*Not at all 1 2 3 4 5 6 7 Very much*

*Degree the 10 values provide motivation to serve self interest.*

**SOR 3. To what degree does each of the following values motivate you to benefit yourself?**

*Strongly disagree* *1 2 3 4 5 6 7 Strongly agree*

1. Making my own decisions about my life.
2. Always discovering new things in life.
3. Enjoying life to the fullest.
4. Striving to be successful and give my best in what I do.
5. Having control over resources and other people.
6. Feeling safe wherever I am.
7. Following social rules out of respect for other people.
8. Following the traditions of my culture.
9. Caring about my family, friends and others around me.
10. Caring about all things on the planet.

*Degree the 10 values motivate to serve other people’s interest.*

**SOR 4. To what degree does each of the following values motivate you to benefit other people?**

*Strongly disagree* *1 2 3 4 5 6 7 Strongly agree*

1. Making my own decisions about my life.
2. Always discovering new things in life.
3. Enjoying life to the fullest.
4. Striving to be successful and give my best in what I do.
5. Having control over resources and other people.
6. Feeling safe wherever I am.
7. Following social rules out of respect for other people.
8. Following the traditions of my culture.
9. Caring about my family, friends and others around me.
10. Caring about all things on the planet.

Degree personal goals serve self interest

**SOR 5. To what degree do your most important goals help you benefit yourself?**

*Strongly disagree* *1 2 3 4 5 6 7 Strongly agree*

1. Making my own decisions about my life.
2. Always discovering new things in life.
3. Enjoying life to the fullest.
4. Striving to be successful and give my best in what I do.
5. Having control over resources and other people.
6. Feeling safe wherever I am.
7. Following social rules out of respect for other people.
8. Following the traditions of my culture.
9. Caring about my family, friends and others around me.
10. Caring about all things on the planet.

*Degree personal goals serve other people’s interest.*

**SOR 6. To what degree do your most important goals help you benefit other people?**

*Strongly disagree* *1 2 3 4 5 6 7 Strongly agree*

1. Making my own decisions about my life.
2. Always discovering new things in life.
3. Enjoying life to the fullest.
4. Striving to be successful and give my best in what I do.
5. Having control over resources and other people.
6. Feeling safe wherever I am.
7. Following social rules out of respect for other people.
8. Following the traditions of my culture.
9. Caring about my family, friends and others around me.
10. Caring about all things on the planet.

## Flexibility

Before you answer the following three questions, please, take a moment to reflect on the current pandemic outbreak and the extent to which this disrupted your most important and meaningful goals. Alternatively, you may think of another situation, which potentially could stop you from pursuing your most important and meaningful goals.

**To what degree are the following statements true to you?**

In a situation where I cannot pursue my most important and meaningful goals...

*Not at all* *1 2 3 4 5 6 7 Very much*

1. I adapt my plans to the new circumstances quite easily.
2. I remind myself that other things in life are just as important.
3. After a serious drawback, I soon turn to new tasks.
4. I prefer to change my goals rather than to keep trying.
5. I have many possible ways of pursuing my goals in any given situation.
6. I am willing to listen and consider alternative ways to pursue my goals.
7. I let go of the goals and stop thinking about them.
8. I seek other meaningful goals.
9. I start working on other new goals.
10. I still try to keep all my goals. (R)
11. I think about what else is important to me.
12. I consider what other goals I could achieve under the circumstances.
13. I think about what exactly I really want.
14. I direct my efforts at what is still possible.
15. I re-define my goals.
16. I reflect on the meaning of the event.
17. I allow myself to experience the painful emotions linked with the event.
18. I change my daily routine.

Appendix B

# **CONSENT FORM**

**Declaration of consent to participate in the study “Whatever makes you happy”**

In this online study, you will answer questions about the things you find most important and meaningful and contribute to your life satisfaction, daily. The study may last approximately 10-15 minutes and you will receive CHF 2 through your Respondi account.

This study is carried out by the Department of Psychology, at the University of Zurich. Anastasia Besika and Mike Martin are responsible for the study. The data collection is completely anonymized, used exclusively for scientific purposes and may be shared with other researchers. The exchange of anonymized data is a common practice that is required for transparent and open research. Participation in this study is voluntary. You can revoke your consent to participate at any time and without giving a reason. There are no known or expected risks resulting from participation in this study. A possible benefit of participation is that you may focus on what matters to you most. The aim of this project is to investigate how people maintain a sense of stability in a constantly changing environment.

If you have any questions about the study, please contact Anastasia at [besika@psychologie.uzh.ch](mailto:besika@psychologie.uzh.ch) or contact the local Ethics Committee of the University of Zurich.

**Declaration of consent to participate in the study “Whatever makes you happy”**

I have read the declaration of consent and would like to participate in the study.

No, I do not want to participate in the study.

**Demographics**

- How old will you be on your next birthday?
- What is your gender?
- What is your ethnicity?
- Did you ever report a psychiatric or a mental health issue such as anxiety?

Appendix C

| **Table 1.**  *Description of samples used for cross-validation.* | | | | |
| --- | --- | --- | --- | --- |
|  | Sample size | Gender | Age | |
|  | (*n*) | Females | *M* | *SD* |
| Total data | *N*= 933 | 467 | 43.51 | 13.42 |
| Sample 1 | 468 | 234 | 43.94 | 13.03 |
| Sample 2 | 465 | 232 | 43.08 | 13.80 |
| Sample 3 | 466 | 0 | 43.43 | 13.10 |
| Sample 4 | 467 | 467 | 43.59 | 13.74 |
| Sample 5 | 311 | 156 | 28.55 | 4.47 |
| Sample 6 | 311 | 155 | 42.75 | 4.92 |
| Sample 7 | 311 | 156 | 59.23 | 4.89 |
| *Note.* *n* = sample size; *M* = Mean; *SD* = Standard Deviation. | | | | |

| **Table 2.**  *Internal consistency results for the multiple-items of the questionnaire.* | | | | | | |
| --- | --- | --- | --- | --- | --- | --- |
| Study 1 | |  |  | |  |  |
|  | | *alpha* | CI | | *omega* | CI |
| C1 | | .76 | .72 - .79 | | .82 | .72 - .79 |
| C2 | | .81 | .78 - .83 | | .86 | .78 - .83 |
| C3 | | .87 | .86 - .89 | | .91 | .86 - .89 |
| C4 | | .82 | .80 - .85 | | .88 | .80 - .85 |
| C5 | | .96 | .96 - .97 | | .97 | .96 - .97 |
| C6 | | .86 | .83 - .87 | | .89 | .84 - .87 |
| SO3 | | .83 | .80 - .85 | | .89 | .80 - .84 |
| SO4 | | .87 | .85 - .88 | | .91 | .85 - .89 |
| SO5 | | .94 | .93 - .95 | | .94 | .94 - .95 |
| SO6 | | .94 | .93 - .95 | | .96 | .93 - .95 |
| Study 2 | | | | | | |
| C1 | .80 | | | .77 - .82 | .85 | .77 - .83 |
| C2 | .81 | | | .79 - .84 | .86 | .79 - .84 |
| C4 | .85 | | | .83 - .86 | .89 | .82 - .86 |
| C6 | .85 | | | .83 - .87 | .90 | .83 - .87 |
| SO3 | .84 | | | .82 - .86 | .90 | .81 - .86 |
| SO4 | .86 | | | .85 - .88 | .91 | .85 - .88 |
| *Note.* In study 1 *n* = 468 and in study 2 *n* = 465; alpha = Cronbach’s *alpha*; *omega* = Revelle’s omega; CI = confidence interval. | | | | | | |

| **Table 3.**  *Eight items were eliminated from the subscales that aimed to assess Consistency and Flexibility to increase Cronbach’s alpha to its maximum.* | | |
| --- | --- | --- |
| Subscale | Item | Item description |
| Flexibility | F4 | I prefer to change my goals rather than to keep trying. |
|  | F7 | I let go of the goals and stop thinking about them. |
|  | F10 | I still try to keep all my goals. *R** |
|  | F17 | I allow myself to experience the painful emotions linked... |
|  | F18 | I change my daily routine. |
| Consistency | C3 | Sometimes I wish I could... *R** |
|  | C5 | Some of my goals stop me from... *R** |
|  | C7 | Seeing other people.... |

*Note.* * Reverse coded item.

| **Table 4.**  *Study 1: Structure matrix from third Exploratory Factor Analysis.* | | | |
| --- | --- | --- | --- |
|  |  | Pattern coefficients | |
| Item | Description | Flexibility | Consistency |
| Flex_9 | I start working on other new goals. | 0.92 | 0.45 |
| Flex_8 | I seek other meaningful goals. | 0.88 | 0.45 |
| Flex_15 | I re-define my goals | 0.75 | .42 |
| C_4 | My most important goals show that... | 0.41 | 0.86 |
| C_2 | In my daily life... | 0.44 | 0.84 |
| C_6 | It is very important (to me) that | 0.39 | 0.70 |
| *Note.* *n* = 468; C = Consistency; F = Flexibility. | | | |

| **Table 5.**  *Study 1: Correlation matrix of the six items retained after the third Exploratory Factor Analysis.* | | | | | | |  |
| --- | --- | --- | --- | --- | --- | --- | --- |
| Item | C_2 | C_4 | C_6 | Flex_4 | Flex_9 | Flex_15 | |
| C_2 | 1 |  |  |  |  |  | |
| C_4 | 0.72 | 1 |  |  |  |  | |
| C_6 | 0.59 | 0.59 | 1 |  |  |  | |
| Flex_8 | 0.38 | 0.36 | 0.35 | 1 |  |  | |
| Flex_9 | 0.39 | 0.34 | 0.33 | 0.81 | 1 |  | |
| Flex_15 | 0.35 | 0.34 | 0.33 | 0.66 | 0.69 | 1 | |
| *Note.* *n* = 468; C = Consistency; F = Flexibility | | | | | | | |

|  |
| --- |

| **Table 6.**  *Study 1: Exlporatory Factor Analysis results on the items assessing Self/Others Ratio.* | | |
| --- | --- | --- |
| Item | Factor | *h^2^* |
| SOR1 | .74 | .55 |
| SOR2 | .72 | .52 |
| SOR3 | .81 | .66 |
| SOR4 | .82 | .67 |
| SOR5 | .75 | .56 |
| SOR6 | .78 | .60 |
| *Note.* SOR = Self/Others Ratio; *h^2^* = communalities. | | |

| **Table 7.**  *Study 2: Structure matrix from Confirmatory Factor Analysis.* | | | |
| --- | --- | --- | --- |
|  |  | Pattern coefficients | |
| Item | Decription | Flexibility | Consistency |
| F9 | I start working on other new goals. | 0.89 | .50 |
| F8 | I seek other meaningful goals. | 0.86 | .51 |
| F15 | I re-define my goals | 0.71 | .42 |
| C4 | My most important goals show that... | .45 | 0.91 |
| C6 | It is very important (to me) that | .50 | 0.79 |
| C2 | In my daily life... | .54 | 0.78 |
| *Note.* *n* = 465; C = Consistency; F = Flexibility. | | | |

**Table 8.**

*Study 2:* *Results* *of Hierarchical Confirmatory Factor Analysis.* (double click on the file name to open)

| **Table 9.**  *Study 2: Confirmatory Factor Analysis (CFA) loadings of items assessing Self/Others Ratio.* | | |
| --- | --- | --- |
|  | CFA | |
| Item | Factor | *h^2^* |
| SOR1 | .65 | .42 |
| SOR2 | .64 | .40 |
| SOR3 | .79 | .63 |
| SOR4 | .81 | .65 |
| SOR5 | .67 | .45 |
| SOR6 | .72 | .51 |
| *Note.* SOR = Self/Others Ratio; *h^2^* = communalities. | | |

| **Table 10.**  *Study 2: Results of* *t-tests comparing mean differences in well-being variable across Self/Others Ratio (within range 0.95-1.05) groups.* | | | | | | | | | | | | | |  |
| --- | --- | --- | --- | --- | --- | --- | --- | --- | --- | --- | --- | --- | --- | --- |
|  | Outside range | | | Within range | | |  | | | |  |  |  | |
| Scale | *n* | *M* | *SD* | *n* | *M* | *SD* | *t* | *df* | *p* | *d* | | | |  |
| MEMS | 262 | 4.17 | 1.37 | 203 | 4.52 | 1.26 | 2.82 | 463 | .005 | .26 | | | |  |
| Satisfaction with Life |  | 4.05 | 1.49 |  | 4.41 | 1.37 | 2.68 |  | .008 | .25 | | | |  |
| Subjective Happiness |  | 4.11 | 1.18 |  | 4.53 | 1.14 | 3.92 |  | .001 | .37 | | | |  |
| Psych. Well-Being |  | 4.61 | 0.94 |  | 4.70 | 0.86 | 1.06 |  | .289 | - | | | |  |
| Perceived Stress |  | 3.92 | 1.29 |  | 3.92 | 1.24 | 0.01 |  | .991 | - | | | |  |
| *Note.* *n* = group size, *M* = mean; *SD* = standard deviation; *df* = degrees of freedom; *d* = Cohen’s *d;* MEMS = Multidimensional Existential Meaning Scale. | | | | | | | | | | | | | |  |

| **Table 11.**  *Study 2:* Significant results of moderation analyses. | | | | | | |
| --- | --- | --- | --- | --- | --- | --- |
|  | MEMS | | | SH | | |
| Predictor | *beta* | *p* | *R^2^* | *beta* | *p* | *R^2^* |
| Consistency | .99 | .001 | .31 | .79 | .001 | .21 |
| Consistency * SOR [1] | .20 | .159 |  | -.31 | .022 |  |
| Flexibility | .66 | .001 | .20 | .57 | .001 | .24 |
| Flexibility * SOR [1] | .24 | .029 |  | -.22 | .084 |  |
| Age | .07 | .280 |  | .20 | .001 |  |
| *Note*. *n* = 465; SOR[1] = Self/Others Ratio within range 0.80 – 1.20. | | | | | | |

| **Table 12**.  *Study 3: alphas and omegas of measures in data subsets.* | | | | | | | | | | |
| --- | --- | --- | --- | --- | --- | --- | --- | --- | --- | --- |
|  | Males | | Females | | 18-34 | | 35-51 | | 52-68 | |
| *Scale* | *α* | *ω* | *α* | *ω* | *α* | *ω* | *α* | *ω* | *α* | *ω* |
| Psychological Balance | .87 | .93 | .82 | .91 | .84 | .93 | .86 | .93 | .84 | .94 |
| Consistency | .88 | .89 | .83 | .84 | .85 | .85 | .88 | .88 | .85 | .87 |
| Flexibility | .87 | .88 | .87 | .88 | .84 | .84 | .86 | .87 | .91 | .91 |
| MEMS | .95 | .97 | .95 | .96 | .94 | .96 | .96 | .97 | .95 | .96 |
| Subjective Happiness | .66 | .69 | .72 | .82 | .71 | .82 | .67 | .80 | .69 | .85 |
| Satisfaction With Life | .92 | .94 | .91 | .93 | .91 | .93 | .93 | .95 | .91 | .93 |
| Psycholog. Well-Being | .89 | .90 | .83 | .86 | .78 | .86 | .86 | .90 | .88 | .90 |
| Perceived Stress | .89 | .93 | .89 | .92 | .86 | .91 | .89 | .94 | .91 | .94 |
| *Notes.* α = Cronbach’s alpha*;* ω *=* Revvell’s omega; MEMS = Multidimensional Existential Meaning Scale. | | | | | | | | | | |

**Table 13.**

*Study 3:* *Overall model fit for males’ data subset.* (double click on the file name to open)

**Table 14.**

*Study 3:* *Overall model fit for females data subset.*

**Table 15.**

*Study 3: Overall model fit summary for age group 18-34 data subsets.*

**Table 16.**

*Study 3: Overall model fit summary for age group 35-50 data subsets.*

**Table 17.**

*Study 3: Overall model fit summary for age group 51-68 data subsets.*

| **Table 18.**  *Results of t-tests comparing gender differences in Consistency and Flexibility.* | | | | | | | | |
| --- | --- | --- | --- | --- | --- | --- | --- | --- |
|  |  | Males | | Females | |  |  |  |
|  | *df* | *M* | *SD* | *M* | *SD* | *t* | *p* |  |
| *Sample 1* | | | | | | | | |
| Consistency | 466 | 5.12 | .90 | 5.21 | .84 | .90 | .368 |  |
| Flexibility |  | 4.44 | 1.38 | 4.61 | 1.21 | 1.39 | .165 |  |
| *Sample 2* | | | | | | | | |
| Consistency | 463 | 5.12 | .94 | 5.29 | .79 | 2.18 | .030* |  |
| Flexibility |  | 4.55 | 1.25 | 4.68 | 1.13 | 1.18 | .238 |  |
| *N = 933* | | | | | | | | |
| Consistency | 931 | 5.11 | .85 | 5.19 | .74 | 1.43 | .154 |  |
| Flexibility |  | 4.51 | 1.31 | 4.64 | 1.17 | 1.83 | .068 |  |
| *Note. n* = sample size; *M* = mean; *SD* = standard deviation; *t* = size of difference;  *df* = degrees of freedom; *p* = significance; * *p* was not significant afer applying Bonferroni correction. | | | | | | | | |

| **Table 19*.***  *Results of muliple comparisons across age groups in Consistency and Flexibility.* | | | | | | | | |
| --- | --- | --- | --- | --- | --- | --- | --- | --- |
|  | *Sample 1* | |  | *Sample 2* | |  | *N = 933* | |
| Age group | Mean difference | *p* |  | Mean difference | *p* |  | Mean difference | *p* |
| *Consistency* | | | | | | | | |
| 35 - 50 / 18 - 34 | -.194 | .121 |  | -.035 | .931 |  | -.139 | .072 |
| 51 - 68 / 18 - 34 | -.022 | .972 |  | -.156 | .254 |  | -.121 | .139 |
| 52 – 68 / 35 - 50 | -.171 | .189 |  | -.121 | .440 |  | -.019 | .953 |
| *Flexibility* | | | | | | | | |
| 35 - 50 / 18 - 34 | -.068 | .888 |  | -.113 | .679 |  | -.093 | .618 |
| 51 - 68 / 18 - 34 | -.275 | .145 |  | -.236 | .190 |  | -.260 | .024* |
| 52 – 68 / 35 - 50 | -.207 | .332 |  | -.122 | .638 |  | -.167 | .214 |
| *Note*. * *p* was not significant afer applying Bonferroni correction. | | | | | | | | |

| **Table 20**  *Study 3: Data subsets based on three Self/Others Ratio ranges.* | | | | |
| --- | --- | --- | --- | --- |
| Range | Sample size | Gender | Age | |
|  | *n* | Females | *M* | *SD* |
| .95 - 1.15 | 820 | 403 | 43.18 | 13.23 |
| .90 - 1.20 | 618 | 307 | 44.92 | 13.41 |
| .80 - 1.20 | 310 | 149 | 42.54 | 13.11 |
| *Note.* *n* = sample size; *M* = mean; *SD* = standard deviation. | | | | |

| **Table 21.**  *Study 3: Exporations of the critical Self/Others Ratio range using the whole data set.* | | | | | | | | | | | | |
| --- | --- | --- | --- | --- | --- | --- | --- | --- | --- | --- | --- | --- |
|  |  |  | Outside range | |  | Within range | | |  |  |  |  |
|  |  | *n* | *M* | *SD* |  | *M* | *SD* | *t* | | *df* | *p* | *d* |
| *Ratio range: 0.95 – 1.05* | | | | | | | | | | | | |
| Consistency | | 820 | 4.94 | 0.86 |  | 5.36 | 0.81 | 7.12 | | 818 | .001 | .49 |
| Flexibility | |  | 4.98 | 1.15 |  | 5.19 | 1.09 | 2.64 | |  | .008 | .18 |
| MEMS | |  | 4.13 | 1.35 |  | 4.64 | 1.21 | 5.67 | |  | .001 | .40 |
| Satisfaction With Life | |  | 3.91 | 1.51 |  | 4.45 | 1.38 | 5.49 | |  | .001 | .38 |
| Subjective Happiness | |  | 4.04 | 1.20 |  | 4.55 | 1.10 | 6.29 | |  | .001 | .44 |
| Psychological Well-Being | |  | 4.67 | 0.88 |  | 4.74 | 0.82 | 1.81 | |  | .238 | - |
| Perceived Stress | |  | 3.79 | 1.23 |  | 3.93 | 1.34 | 1.55 | |  | .123 | - |
| *Ratio range: 0.90 – 1.10* | | | | | | | | | | | | |
| Consistency | | 618 | 4.87 | 0.97 |  | 5.24 | .84 | 5.02 | | 622 | .001 | .40 |
| Flexibility | |  | 4.93 | 1.29 |  | 5.10 | 0.98 | 2.81 | |  | .003 | .22 |
| MEMS | |  | 3.98 | 1.46 |  | 4.57 | 1.18 | 5.56 | |  | .001 | .44 |
| Satisfaction With Life | |  | 3.69 | 1.57 |  | 4.37 | 1.41 | 5.60 | |  | .001 | .45 |
| Subjective Happiness | |  | 3.94 | 1.29 |  | 4.43 | 1.11 | 5.14 | |  | .001 | .41 |
| Psychological Well-Being | |  | 4.63 | .94 |  | 4.78 | 0.81 | 2.12 | |  | .034 | .17 |
| Perceived Stress | |  | 3.78 | 1.23 |  | 4.02 | 1.38 | 2.27 | |  | 023 | .18 |
| *Ratio range: 0.80 – 1.20* | | | | | | | | | | | | |
| Consistency | | 310 | 4.79 | 1.03 |  | 5.15 | .88 | 3.32 | | 308 | .001 | .38 |
| Flexibility | |  | 4.83 | 1.54 |  | 5.15 | .99 | 2.24 | |  | .026 | .25 |
| MEMS | |  | 3.91 | 1.55 |  | 4.33 | 1.28 | 2.69 | |  | .008 | .30 |
| Satisfaction With Life | |  | 3.59 | 1.66 |  | 4.28 | 1.50 | 3.81 | |  | .001 | .43 |
| Subjective Happiness | |  | 3.87 | 1.37 |  | 4.28 | 1.11 | 2.90 | |  | .004 | .33 |
| Psychological Well-Being | |  | 4.59 | 1.03 |  | 4.71 | 0.84 | 1.67 | |  | .243 | - |
| Perceived Stress | |  | 3.95 | 1.51 |  | 3.95 | 1.27 | 0.03 | |  | .977 | - |
| *Note. n* = sample size; *M* = mean; *SD* = standard deviation; *df* = degrees of freedom; *d* = Cohen’s *d;* MEMS = Multidimensional Existential Meaning Scale. | | | | | | | | | | | | |

Appendix D

# **PSYCHOLOGICAL BALANCE SCALE**

**Instructions**

Please, answer the following questions intuitively. There are no right or wrong answers. Some questions may sound similar. Please, answer them without worrying about your previous responses.

## Consistency

Values refer to ideals, which may motivate and guide people in life. Values typically represent what is most important to people. We are using ten sentences that describe ten social values to help us understand what you value most in life.

**C1** (Degree daily actions express personal values)

**To what extent do you agree with the following statements?**

*Strongly disagree* *1 2 3 4 5 6 7 Strongly agree*

**In my daily life...**

1. I decide about what I do.
2. I can discover new things.
3. I enjoy the day to the fullest.
4. I give my best in what I do and strive to succeed.
5. I strive to gain resources and influence others.
6. I make sure I am safe wherever I am.
7. I follow social rules out of respect for other people.
8. I follow the traditions of my culture.
9. I show that I care about my family, friends and others around me.
10. I show that I care about all things on the planet.

**C2** (Degree most important personal values guide important goals)

**Thinking of your most important personal goals altogether, to what extent do you agree with the following statements?**

*Strongly disagree* *1 2 3 4 5 6 7 Strongly agree*

**My most important goals show that:**

1. I decide about which way my life goes.
2. I am open to discovering new things in life.
3. I can enjoy life to the fullest.
4. I strive to do my best and succeed in what I do.
5. I strive to gain resources and influence others.
6. I want to be safe wherever I am.
7. I can follow social rules out of respect for others.
8. I can follow ideas of my culture or religion.
9. I care about my family, friends and other people.
10. I care about all things on the planet.

**C3** (Degree of value salience)

**Over the last week, how often did you think in this (or a similar) way?**

*Never* *1 2 3 4 5 6 7 Very often*

**It is very important (to me) that:**

1. I make my own decisions about my life.
2. I can discover new things in life.
3. I enjoy life to the fullest.
4. I give my best in what I do and succeed.
5. I have resources and can influence others.
6. I feel safe in my environment and in myself.
7. I show respect to others by following social rules.
8. I follow the traditions of my culture.
9. I care about my family, friends and others around me.
10. I protect all things on earth.

## Flexibility

**Instructions**

Before you answer the following three questions, please, take a moment to reflect on a situation that could disrupt your most important and meaningful goals.

**To what degree are the following statements true to you?**

In a situation where I cannot pursue my most important and meaningful goals...

*Not at all* *1 2 3 4 5 6 7 Very much*

1. I seek other meaningful goals.
2. I start working on other new goals.
3. I re-define my goals.

## Self/Others Ratio (SOR = SOR 1 / SOR 2)

**SOR 1** (Degree the 10 values provide motivation to serve personal interest)

**To what degree does each of the following values motivate you to benefit yourself?**

*Strongly disagree* *1 2 3 4 5 6 7 Strongly agree*

1. Making my own decisions about my life.
2. Always discovering new things in life.
3. Enjoying life to the fullest.
4. Striving to be successful and give my best in what I do.
5. Having control over resources and other people.
6. Feeling safe wherever I am.
7. Following social rules out of respect for other people.
8. Following the traditions of my culture.
9. Caring about my family, friends and others around me.
10. Caring about all things on the planet.

*Degree the 10 values motivate to serve other people’s interest.*

**SOR 2. To what degree does each of the following values motivate you to benefit other people?**

*Strongly disagree* *1 2 3 4 5 6 7 Strongly agree*

1. Making my own decisions about my life.
2. Always discovering new things in life.
3. Enjoying life to the fullest.
4. Striving to be successful and give my best in what I do.
5. Having control over resources and other people.
6. Feeling safe wherever I am.
7. Following social rules out of respect for other people.
8. Following the traditions of my culture.
9. Caring about my family, friends and others around me.
10. Caring about all things on the planet.
